# Mapping and assessing ecosystem services for sustainable policy and decision-making in Eritrea

**DOI:** 10.1007/s13280-023-01841-4

**Published:** 2023-03-18

**Authors:** Blal Adem Esmail, Chiara Cortinovis, Jingxia Wang, Davide Geneletti, Christian Albert

**Affiliations:** 1 Institute of Geography, Ruhr University Bochum, Ruhr University Bochum, Universitätsstr. 150, 44805 Bochum, Germany; 2 Department of Geography, Humboldt-Universität Zu Berlin, Rudower Chaussee 16, 12489 Berlin, Germany; 3 Department of Urban Studies and Planning, The University of Sheffield, Western Bank, Sheffield, S10 2TN UK; 4 Department of Civil, Environmental & Mechanical Engineering, University of Trento, 38123 Trento, Italy

**Keywords:** East Africa, Global Copernicus Land cover, IPBES, Land cover change analysis, Matrix approach, SEEA-EA

## Abstract

The mapping and assessment of ecosystems and their services (MAES) is key to inform sustainable policy and decision-making at national and sub-national levels. Responding to the paucity of research in sub-Saharan Africa, we conduct a pilot study for Eritrea that aims to map and assess the temporal dynamics of key ecosystems and their services. We reviewed policy and legal documents, analyzed land cover changes and estimated the potential for ecosystem services supply through an expert-based matrix approach. Our results showed that from 2015 to 2019, the potential supply of the ecosystem services analyzed (e.g., crop provisioning, water supply and recreation) increased, with the exception of wood supply. Overall, our study presents policy-relevant insights as to where to conserve, develop, or restore ecosystem services supply in Eritrea. Our approach is transferable to similar data scarce contexts and can thereby support policies toward more sustainable land development for people and nature.

**Supplementary Information:**

The online version contains supplementary material available at 10.1007/s13280-023-01841-4.

## Introduction

Mapping and assessment of ecosystems and their services (MAES) is widely recognized as a crucial step toward sustainable policies and decisions that promote human well-being and preserve life-sustaining ecosystems (MA 2005; Maes et al. 2012; Geneletti et al. 2020). Globally, the need to ensure a sustainable supply of ecosystem services (ES) fostered the creation of the Intergovernmental Science-Policy Platform on Biodiversity and Ecosystem Services (IPBES) (Díaz et al. 2015), and more recently, the adoption of ecosystem accounting standards as part of the United Nations System of Environmental Economic Accounting (SEEA-EA 2021). In Europe, the advancement of MAES has been an essential step toward achieving the targets of the EU Biodiversity Strategy 2020 and 2030 (European Commission 2013; European Commission 2020). Accordingly, European research on MAES concepts and methods has evolved significantly (Maes et al. 2016), for example, through proposed classifications (Haines-Young and Potschin 2013), national assessments (Schröter et al. 2016) and more focused research projects at national level (UK National Ecosystem Assessment 2014; Hermes et al. 2021).

In Africa, better information on ecosystems and their services is much needed to enhance the understanding of current challenges and to outline more sustainable pathways for future development (IPBES 2018; Jamouli and Allali 2020). Climate change and rapid urbanization increasingly threaten the conservation and sustainable use of ES upon which many African societies directly depend. In this context, MAES studies could support more sustainable decision-making and foster transformative change (Archer et al. 2021), ultimately helping to implement the African Union’s vision of an integrated, prosperous, and peaceful Africa by 2063 and associated Sustainable Development Goals (SDGs) and Aichi Biodiversity Targets (IPBES 2018).

MAES research in Africa can build upon an increasing number of studies, primarily from South Africa, Kenya, and more recently Ethiopia (Wangai et al. 2016; Jamouli and Allali 2020; Mekuria et al. 2021). Several studies have applied biophysical assessments of ES at sub-national scales, for example, studies in South Africa (Petz et al. 2014), in Ghana and Côte d'Ivoire (Leh et al. 2013), and in Tanzania by (Fisher et al. 2011; Swetnam et al. 2011). Thematic studies have focused, among others, on potential impacts of land cover changes on ES (Reyers et al. 2009), links between biodiversity and the provision of ES (Naidoo et al. 2011), climate regulation services in cities (Cavan et al. 2014), and synergies between watershed investments, urban water security, and rural poverty alleviation (Adem Esmail and Geneletti 2017). Methods applied in ES valuation include economic approaches (Dumenu 2013; Turpie et al. 2017; Mulatu 2022) and participatory methods (e.g., Fagerholm et al. 2012; Koko et al. 2020). One of the prominent ES mapping studies was presented by Egoh et al. (2008), focusing on five selected ES in South Africa. Noteworthy is the growing number of studies from Ethiopia, including Mekuria et al. (2021) who analyzed changes in land use and land cover based on Landsat imagery and applied value transfer assessment methods to estimate associated losses in ecosystem services values (Mekuria et al. 2021). Similarly, Tolessa et al. (2017a) and Tolessa et al. (2017b) considered satellite imagery for land use change detection as a basis for ES valuation.

Despite this emerging literature, comprehensive approaches to MAES that address multiple ES, especially at the national level, are still rare. Knowledge gaps exist on how MAES could be implemented in the African context and how to design MAES assessments to best inform policy processes. Among others, questions remain on which ES should be prioritized in the analysis, how to address potential data shortages, what scale is the most suitable to support decision-making, and what policies could benefit from the results.

The country of Eritrea, located on the Horn of Africa, represents a suitable case study for advancing MAES approaches and methods, as it offers a diverse set of ecosystems and typical challenges of data scarcity in a relatively small spatial context. Land degradation and desertification are the country’s most serious and widespread environmental problems, compounded by rapid urbanization and the effects of climate change (Murtaza 1998; Burkhard and Maes 2017; Measho et al. 2019; Adem Esmail and Geneletti 2020). Accordingly, national-level policies emphasize sustainable land and water management, promoted through an ambitious greening and ecological restoration campaign and related soil and water conservation measures.

The aim of this pilot study is to map and assess the recent temporal dynamics of key ecosystems and their services in Eritrea to support policy and decision-making at national and sub-national levels. We address three research questions: (i) What is the relevant institutional, policy, and legal context for a MAES application in Eritrea? (ii) How have Eritrean ecosystems changed recently? (iii) What impact might the changes have had on the potential supply of key ES? Our assessment of yearly changes in ecosystems and their services focuses on the national, regional, and sub-regional levels. Based on the results, we will reflect upon implications for policy and decision-making.

## Study area

**Fig. 1:**
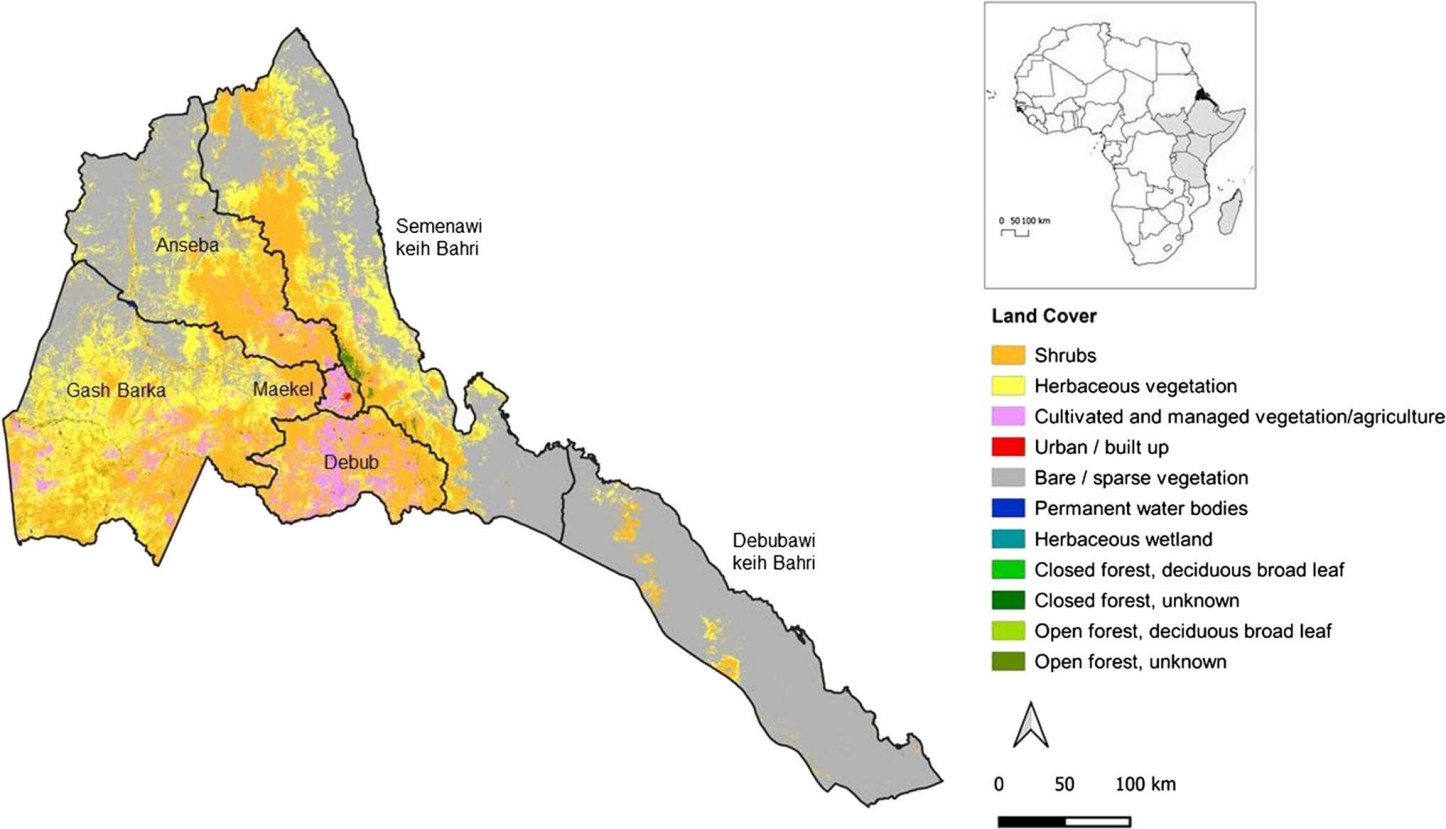
Location of Eritrea in the broader East Africa Region and map of land cover in 2019 in the six regions of the country. Data source: Copernicus Global Land Service (Buchhorn et al. 2020)

Eritrea is located on the Horn of Africa (Fig. 1) and belongs to the East Africa and Adjacent Islands sub-region, which is characterized by a subtropical climate (IPBES 2018). It has an area of about 117,600 km^2^, a coastline along the Red Sea of about 1,720 km, of which about 1,155 km along the mainland coast, and about 565 km around about 350 islands (Tsehaye and Nagelkerke 2008). About half of the territory is bare or covered by sparse vegetation, 43% by shrubs or herbaceous vegetation and just over 5% is cultivated (Buchhorn et al. 2020). Eritrea had a resident population of 3.2 million in 2010 (NSO Eritrea and Fafo AIS 2013). With almost 60% of the population living in rural areas, it is one of the last countries facing rapid urbanization. According to the 2018 World Urbanization Prospects, from 1988 to 2018, the urban population experienced dramatic growth from 17.6% to 40.1% of the total population in just three decades, mostly in the capital Asmara.

The country has a four-tier system of governance, which includes a national, regional (*Zoba*), sub-regional (*Sub-Zoba*) and community (*Kebabi*) level. It is divided into six administrative regions, namely *Anseba*, *Debub*, *Debubawi Keih Bahri, Gash Barka*, *Maekel*, and *Semenawi Keih Bahri*, and 57 sub-regions (see the Supplementary Material—Table S1 and Figure S1). The regions roughly correspond to the country’s major river basins; while their population ranges from 1.4 million in the Debub region to 398,000 in the arid Debubawi Keih Bahri region. In this study, two regions with some distinctive features that make them interesting for exploring the sub-national implications of MAES are the Maekel region and Gash Barka region. The former includes the capital Asmara and is the smallest and most densely populated region, covering less than 1.2% of the total area but housing almost 17% of the total population. The second is the largest region (divided into 14 sub-regions) and includes both a biodiversity hotspot and the country’s most fertile area for agriculture.

Among the projects and initiatives for land and water restoration and conservation promoted by the national government (see Table S13 and Box S1 in Supplementary Material) are about 785 large and small dams that contribute significantly to ensuring water and food security in the country (Ministry of Information 2021, citing the Cartography and Information Center). An arid and semi-arid country, Eritrea has no significant water resources and is threatened by recurrent droughts and desertification. Dams and the water stored in them therefore play a key role in meeting the basic needs of populations in both urban and rural areas. Moreover, Eritrea has established four protected areas, including two national parks (Dahlak Marine National Park and Semenawi Bahri National Park) and two nature reserves (Gash-Setit, and Yob Wildlife Reserve). According to the Forest and Wildlife Authority, the total protected area is 396,930 ha out of around two million hectares planned to be enclosed in future, to promote natural regeneration of trees and grasses.

## Materials and methods

This study follows the tiered approach to mapping and assessing ES (Grêt-Regamey et al. 2015), with the tiers representing different levels of data integration and modeling complexity. Our study is associated with the coarsest level of analysis (tier 1, see also Burkhard et al., (2010)), where ES assessment is mainly based on land cover types, following the approach by Cabral et al., (2021) for a national MAES in Portugal. While this coarseness inhibits input into local land use decisions, it is considered sufficient for country-level estimates of the potential supply of ES and their spatial and temporal distribution. Our study is therefore a first step toward raising awareness of some challenges related to ecosystems and their services.

**Fig. 2:**
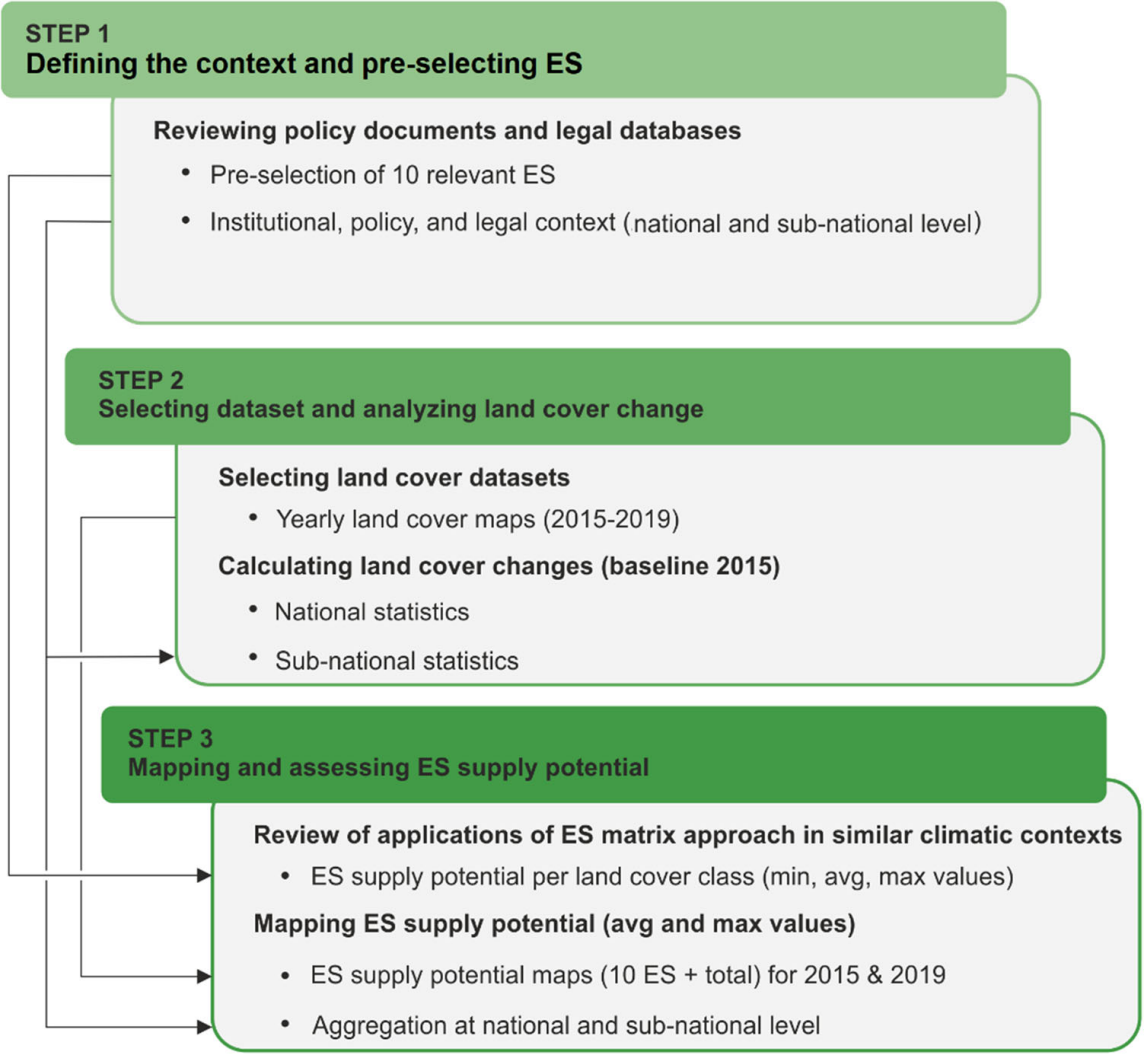
Flowchart of the 3-step method used in the study

Our research design includes three main steps corresponding to the three research questions (Fig. 2). First, we review relevant documents and databases to define the institutional, policy, and legal context and identify key ES to be analyzed. Second, we select landcover data relevant to ES and analyze land cover changes over the period of 2015–2019. Third, we map and assess the ES supply potential and discuss the results in light of the policy priorities in the country. We use free and open source software QGIS v.3.16.8 and R (R Core Team 2020) for spatial and statistical analysis.

### Step 1: defining the context and pre-selecting ES

To effectively support decision-making, MAES applications should be guided by criteria of credibility, saliency, and legitimacy (Cash et al. 2003; Adem Esmail et al. 2017). ES indicators, in particular, are more likely to be useful for policy-making when their audiences simultaneously perceive the indicators and the process of their development as scientifically adequate (credible), relevant to a particular policy objective (salient), politically fair (legitimate), and applicable in practical assessments and monitoring (feasible) (van Oudenhoven et al. 2018). Therefore, we reviewed national policy documents and databases to define the institutional, policy, and legal context in Eritrea and identify the relevant actors. Furthermore, the review of the policy documents served to select key ES to be analyzed.

We started by exploring existing studies and databases useful for characterizing the country’s policies. Existing studies include the work of Habtezion (2015) who critically analyses the Eritrean Water Proclamation No. 162/2010 and Andemariam (2019) who reports on the Cultural and Natural Heritage Proclamation of Eritrea. Another source is Erilaw.com, an online legal search database that contains a comprehensive list of Proclamations and Legal Notes since the country’s independence in 1991. The database lists 261 entries up to 2017, 35 of which could be considered potentially relevant for biodiversity issues; however, it does not propose any thematic organization nor is it freely accessible. In contrast, the FAO Lex database is a comprehensive and up-to-date database, one of the world's largest online repositories of national laws, regulations and policies on food, agriculture, and natural resources management. Of note are also national efforts to compile a comprehensive database of laws and policies and to raise public awareness, such as the recent Law Week 2021, held during December 6–11 across the country (Tesfamichael 2021).

Two key documents were analyzed, namely the 2015 National Biodiversity Strategy & Action Plan (2014–2020) and the 2018 Nationally Determined Contributions (NDCs) to the United Nations Framework Convention on Climate Change—UNFCCC. They formed the basis for the identification and characterization of the main governmental and non-governmental actors and key policy documents related to biodiversity issues and natural resource management in general. The initial list of selected documents was reviewed by a local expert, who suggested additional documents (e.g., the National Charter). The identified policies, grouped into thematic areas (i.e., national policy documents, biodiversity, land degradation, climate change and energy, and cross-cutting areas), were then reviewed to define their focus and extract a brief description (see Supplementary Material—Table S2).

The review of the documents led to the pre-selection of ten ES that are potentially the most relevant to policy and decision-making: four provisioning (i.e., crop, grazed biomass, wood, and water supply), four regulating and maintenance (i.e., global climate regulation, soil and sediment retention, pollination, nursery population, and habitat), and two cultural services (i.e., recreation-related services including physical and psychological experiences, and spiritual, artistic and symbolic services, also relating to supporting identities, cf. Díaz et al. 2018). The services have been classified according to the scheme developed by the United Nations Statistics Division as part of the System of Environmental Economic Accounting—Ecosystem Accounting (SEEA-EA 2021)—see Table S3.

### Step 2: selecting the dataset and analyzing land cover change

We used the Copernicus Global Land Cover (CGLC) version 3.0 with 100 m spatial resolution (Buchhorn et al. 2020) due to its consistency in classification over years and an overall mapping accuracy of more than 80%. The CGLC V3.0 product provides the annual global land cover maps and cover fraction layers for the reference years 2015 to 2019. The relevant 20 × 20 degree ‘E040N20’ tiles, for the 2015–2019 five years period, were downloaded from https://land.copernicus.eu/global/lcviewer and clipped using the national boundary retrieved from the FAO Map Catalogue. An overview of the geodata used in this study can be found in the Supplementary Material—Table S4.

We analyzed land cover changes at the national scale for the four years between 2016 and 2019, using 2015 as the baseline and following the analytical steps as described in ESCAP Statistics Division (ESCAP Statistics Division 2020). For each year, we produced matrices of land cover change transition and a table of percent land cover change relative to 2015 at the national and sub-national level. In addition, we calculated zonal statistics for the five land cover maps (i.e., years 2015–2019) at the national, regional, and sub-regional levels.

### Step 3: mapping and assessing ES supply potential

We conducted a review of selected case studies using the so-called ES matrix approach (Burkhard et al. 2010), in order to determine the potential ES supply by the 11 land cover classes mapped for Eritrea. The matrix approach considers land cover classes as the units of analysis and quantifies their potential to supply each ES through a biophysical value or—more frequently—a score usually ranging from 0 to 5. To define the scores, we started from a sample of 109 studies applying the matrix approach identified by Campagne et al. (2020). We selected the articles that addressed similar climatic conditions as in our case study. Based on global climate classification maps (Beck et al. 2018), we identified ten applications from: Kenya (4), Uganda (1), Burkina Faso (1), South Africa (1), Mexico (2), and USA (1). For each study, we extracted information on location, spatial extent, climatic region, ecosystem/land use classification, ES classification, and number of ES analyzed. We also identified the key methodological aspects such as scoring systems, actors involved, approaches to gathering expert and/or stakeholder opinion, and ES supply potential values attributed to different types of land cover (see Supplementary Material—Table S8). This allowed us to create a database with ranges of ES supply potentials of different land cover classes for the application of the matrix approach in subtropical climatic conditions.

In general, regulating and maintenance services are analyzed most frequently (48), followed by provisioning (42) and cultural services (20). With the exception of two publications (i.e., Martínez-Harms et al. 2016; Sinare et al. 2016), which quantify the services using actual quantities, the remaining eight studies use a 0–5 Likert scale to assess potential ES supply by different land cover types. Thus, for each land cover class, we calculated the maximum, minimum, and average value of potential ES supply based on the eight studies reviewed (see Supplementary Material—Table S8). Notably, the identified cases used different land use classification and ES. Therefore, we ‘translated’ all land covers into broader land cover classes (e.g., cropland, grassland, settlement) and reclassified the ES according to SEEA-EA. We consider both maximum and average literature values to identify initial estimates and to simulate the perspectives and opinions of different stakeholders. However, for the sake of brevity, here we mainly discuss the results in terms of the maximum potential value (Table 1). Our choice had a precautionary basis, i.e., we wanted to avoid overlooking areas of potentially high value, recognizing that further research is needed to validate the values from the literature as local experts’ opinions and/or the actual supply could be significantly different. In the Supplementary Material, we present additional results based on maximum and average values.

**Table 1:**
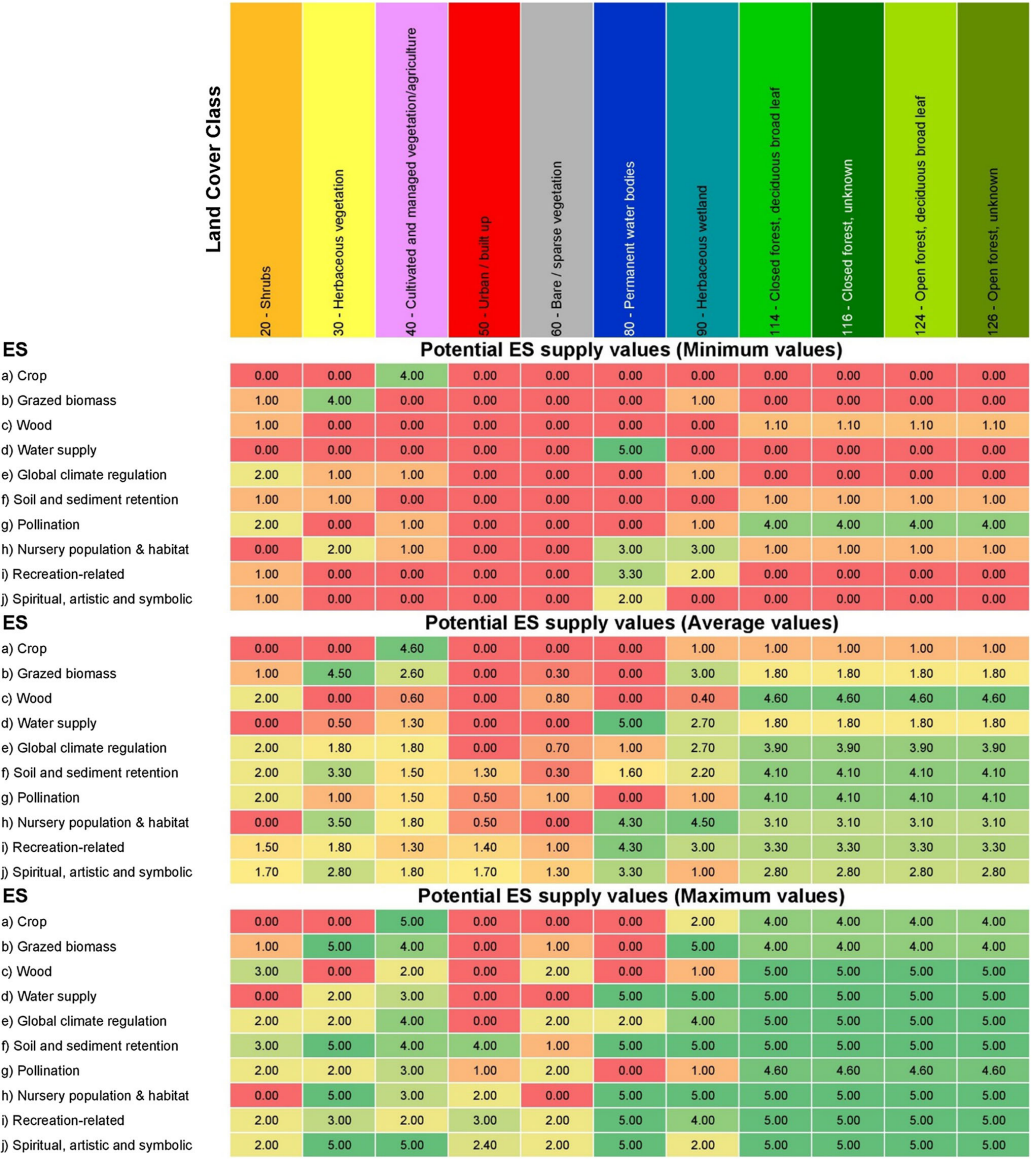
Potential ES supply values (from 0—red to 5—green) assigned to different land cover classes in Eritrea. The values are based on a review of applications of the matrix approach in similar climatic contexts. The land cover classes (including code and name) are based on the Land Cover Classification System (LCCS) developed by the United Nations (UN) Food and Agriculture Organization (FAO). (In this study, both the average and maximum potential values are considered)

We produced 40 maps using both maximum and average values of the potential ES supply of the 11 land cover classes for the years 2019 and 2015 and aggregated them with equal weights to each ES. Four maps of ES supply potential were generated by summing the scores obtained for each ES and then standardizing the result. For the two years, the procedure was repeated for both the maximum and the average ES supply potential values.

The ES maps were individually analyzed to produce zonal statistics according to administrative boundaries. The analysis was performed for the national, regional, and sub-regional boundaries, considering both maximum and average potential ES supply values from Table 1.

## Results

### Institutional, policy, and legal context

An overview of the institutional context in Eritrea in relation to MAES and more generally to biodiversity and natural resource management issues is given in Table 2 (see also Figure S1 in Supplementary Material). According to the documents reviewed, the main governmental actors are the Ministry of National Development, the Ministry of Land, Water and Environment and in particular its Department of Environment, the Forestry and Wildlife Authority, the Ministry of Agriculture, and the Ministry of Marine Resources. However, according to Proclamation 86/1996, actual implementation is mainly the responsibility of regional administrations with the coordinating role of the Ministry of Local Government. Of note is the potential role of non-governmental actors such as community-based and civil society organizations at all levels, including the National Union of Eritrean Women, the National Union of Eritrean Youth and Students, the Dairy, and Horticultural Development Cooperatives, and Water user associations.

**Table 2:** Key governmental actors in the four tiers of governance in Eritrea. * Actors responsible for the bulk of issues concerning biodiversity, and natural resource management in the country

Tier	Governmental actors
National	Ministry of National Development (MoND)*
	Ministry of Land, Water & Environment (MoLWE)*
	Department of Environment (DoE) of the MoLWE*
	Forestry and Wildlife Authority (FWA)*
	Ministry of Agriculture (MoA)*
	Ministry of Marine Resources (MoMR)*
	Ministry of Energy and Mines
	Ministry of Local Government (MoLG)
	Ministry of Information (MoI)
	Ministry of Education (MoE)
Regional	Regional administration
	MoLG in collaboration with MoA and FWA
	Department of Agriculture & Land of Regional Adm
Sub-regional	Sub-Regional Administration
Community	Local administration

In terms of policy and legal context, in addition to the Constitution, we identified 54 relevant policy documents related to biodiversity conservation issues, grouped into five thematic areas, including National Policy Documents, Biodiversity, Land Degradation, Climate Change and Energy, and seven cross-cutting thematic areas. The National Biodiversity Strategy and Action Plan (2015) envisions that “by 2040, biodiversity is valued, conserved, restored, and wisely used through the active participation of all stakeholders to sustain a healthy environment and equity sharing of benefits to meet the development needs and well-being of the people.” Further important policy documents with relevance for biodiversity and ES are the report on Nationally Determined Contributions (NDCs) to the UNFCCC of 2018, and the National Charter (1994), outlining six policy objectives and guiding principles for the future of Eritrea. An overview of the policy and legal context is presented in the Supplementary Material—Table S2.

### Changes in Eritrean ecosystems in 2015–2019

#### National land cover change statistics

The two land cover classes showing the greatest changes are Herbaceous vegetation (gain) and Bare/sparse vegetation (loss). This is highlighted in Table 3, which depicts the land cover change matrix between 2015 and 2019. For more detailed results of the changes, see Table S5 in the Supplementary Material.

**Table 3:**
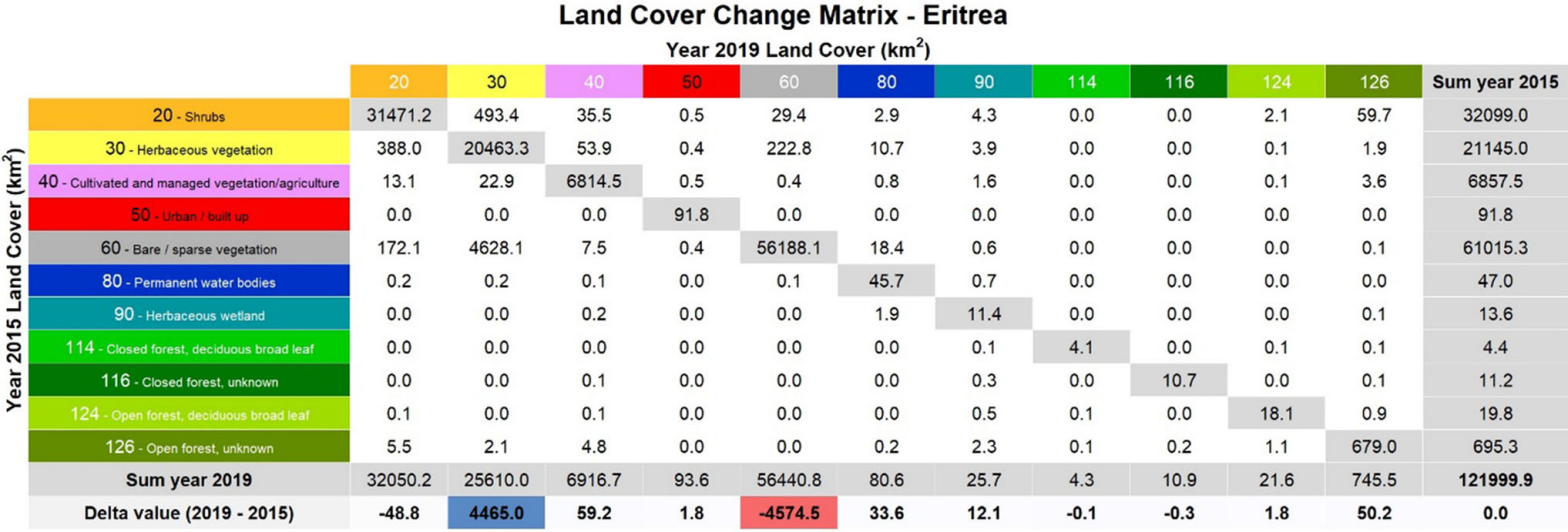
Land cover change matrix for the years 2015 and 2019. Each cell shows the transition from the land cover class in the row (identified by a code and a name) to the land cover class in the column (identified by a code). The areal extent of each land cover class in 2015 is represented in the last column, while the last rows represent the extent in 2019 and the changes compared to 2015 (blue color for gains and red color for loss)

As shown by Table 3, taking the year 2015 as a reference, the Bare/sparse vegetation class has the largest share (61,015 km2 or 50% of the total), followed by Shrubs (32,099 km2 or 26.6%), Herbaceous vegetation (21,145 km^2^ or 17.3%) and Cultivated and managed vegetation/agriculture (6858 km2 or 5.6%). The remaining land cover classes are all less than 1%, including Open Forests (695 km2 or 0.6%) and Urban/Built up areas (92 km2 or 0.1%). A similar percentage distribution of the various land covers is also observed for 2019, with the exception of Herbaceous vegetation and Bare/sparse vegetation. In absolute terms, the largest gain is seen in Herbaceous vegetation (+ 4,465 Km^2^), while in percentage terms, the Herbaceous wetland increases the most (+ 88%). The land cover class Bare/sparse vegetation shows the highest losses anyway (− 4,575 Km^2^). Notable is a sudden change after 2017, with a significant increase in Herbaceous vegetation versus a decrease in Bare/sparse vegetation. In fact, all of the above considerations require possible explanations, which could come either from what has happened on the ground (e.g., policies, plans, and projects implemented) or from inaccuracies in the land cover data used.

#### Sub-national land cover change statistics

At a regional level, our analysis revealed that in 2019 the largest increase for Herbaceous vegetation was recorded in the regions of Semenawi Keih Bahri (+ 2,905 km^2^) and Anseba (+ 1,340 km^2^). This contrasts with the largest decline in the Bare/Sparse vegetation of 3041 km^2^ and 1228 km^2^, respectively, in the same two regions. The largest percentage change is recorded for the class Herbaceous wetland in the Maekel region (+ 429%), Permanent water bodies in the Anseba region (+ 129%) and Herbaceous vegetation in the Semenawi Keih Barhi region (+ 60%). Note that the large percentages usually correspond to land cover classes that cover only a small part of the region. Our findings focusing on the Maekel region showed that the largest recorded change is less than 1.4 km^2^. In particular, no changes are registered for three forest classes (114, 116, and 124) vis-à-vis a modest increase in Urban/built, Cultivated areas, and Open forests (126), and a decrease in Shrubs and Herbaceous vegetation. In the Gash Barka region, on the other hand, an increase in Herbaceous vegetation (+ 195 km^2^), Permanent water bodies (+ 11 km^2^), Herbaceous wetland (+ 5.4 km^2^), and Open forests (+ 4 km^2^) is observed against a significant decrease of Bare/Sparse vegetation (− 188 km^2^) and Shrubs (− 0.2 km^2^). Notably, Herbaceous wetlands and Permanent water bodies saw the highest percentage increases. Detailed results on regional land cover changes are reported in Table S6 in the Supplementary Material.

Similarly, our analysis revealed the main land use changes registered at the sub-regional level, here presented for two illustrative regions, Gash Barka and Maekel. In absolute terms, some sub-regions of the Gash Barka have seen an increase in herbaceous vegetation at the expense of bare soil (e.g., in Forto), while other sub-regions have experienced percentage increases in herbaceous wetlands and permanent water bodies (e.g., Tesseney, La'elay Gash, and Ombager). Also, of note is the loss of forest in La'elay Gash, Forto and Haikota. Similarly, in the Maekel region, some sub-regions recorded an increase in open forests and herbaceous wetlands in absolute terms at the expense of shrubs (e.g., Serejeqa), while other sub-regions recorded percentage increases in herbaceous wetlands and permanent water bodies (e.g., Berik). Detailed results on sub-regional land cover changes for all six regions are reported in Table S7 in the Supplementary Material.

### Mapping and assessment of ES supply potential

#### Mapping of selected ES

**Fig. 3:**
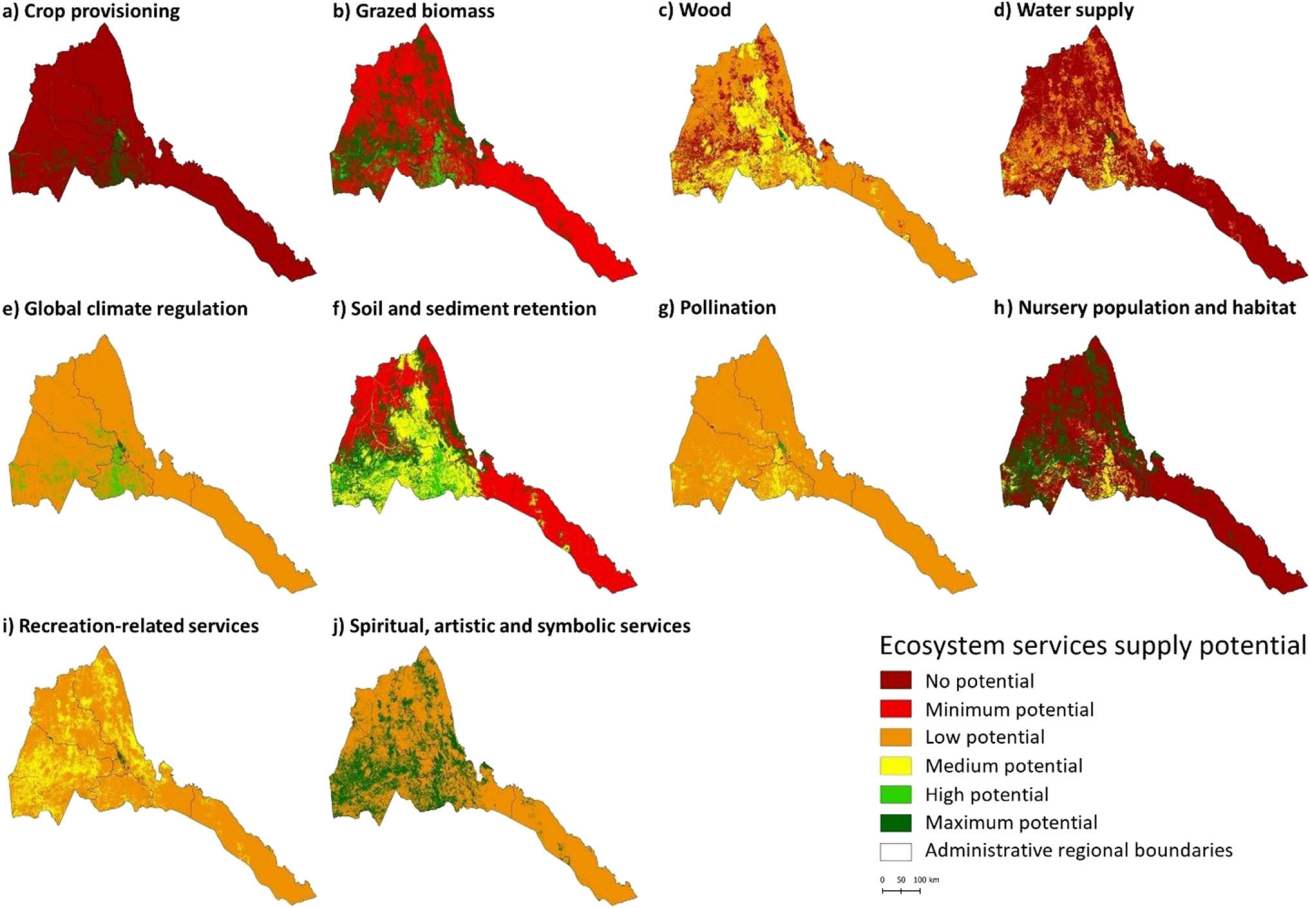
Maps of ES supply potential in Eritrea for 2019 based on the maximum values obtained from literature review (see Table 1)

**Fig. 4:**
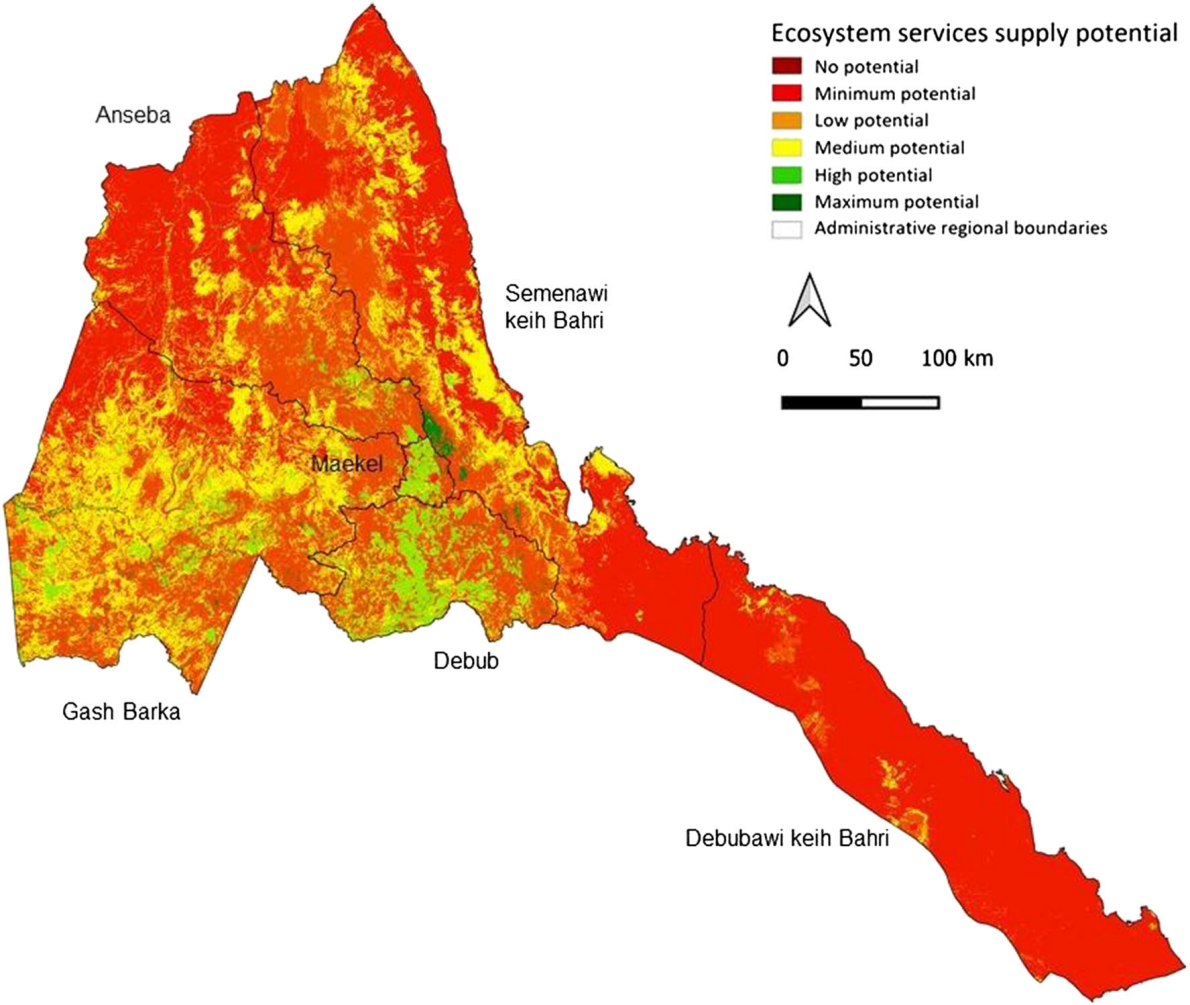
Map of overall ES supply potential in Eritrea for 2019 based on the maximum values obtained from the literature review (see Table 1)

The maps of ES supply potential are shown in Fig. 3. An overall potential ES supply map for the year 2019 is shown in Fig. 4. In general, all maps refer to the maximum potential ES supply values in Table 1; similar maps based on the average potential values are presented in the Supplementary Material (Figures S2, S3 and S4).

#### Aggregated ecosystem service supply potential values

Considering the maximum potential values from the literature, the services with the highest average values per unit area (Table 4) are spiritual, artistic, and symbolic services (j), soil and sediment retention (f) and recreation services (i). The services with the lowest values are—starting with the last one—water supply (d), crop provisioning (a), and nursery population and habitat (h). Additionally, between 2015 and 2019, the largest increases were recorded for Nursery population and habitat (+ 0.19), and for soil and sediment retention and grazed biomass (both + 0.15). The overall mean value increased by 0.07. Detailed results on national aggregation, considering both maximum and average literature values, are reported in Table S9 in the Supplementary Material

**Table 4:**
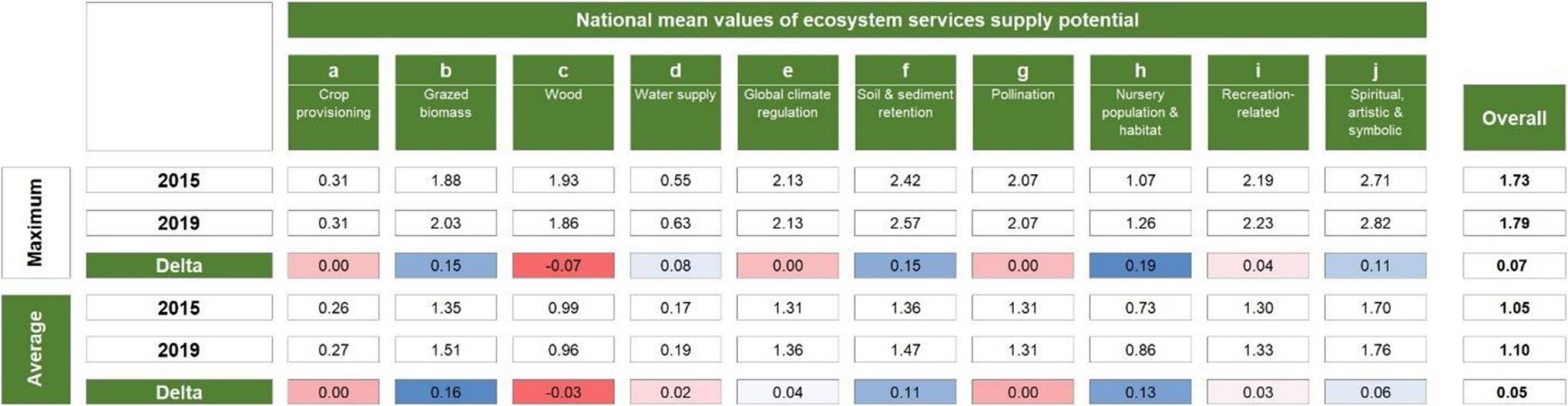
Average ES supply potential at the national level and its changes over the period 2015 to 2019, considering the maximum and average values from the literature (see Table 1). For each ES, the national mean value per unit area is reported for the years 2015 and 2019, and in terms of change (highlighted in a red to blue color scale)

Potential ES supply per unit area differed across regions (Table 5). Notable are the generally lower values in Debubawi Keih Bahri compared to the Maekel and Gash Barka regions. In terms of changes, the mean values of the potential ES supply increased for all considered services and regions from 2015 to 2019. The only exception is wood supply (c), which mainly decreased in the regions of Semenawi Keih Bahri (− 0.17) and Anseba (− 0.14). Overall, the two regions with the highest increase are Semenawi Keih Bahri and Anseba, while the Debub region has the lowest increase. Detailed results on the regional aggregation of potential supply of ES and their changes, considering maximum literature values, are reported in Table S10 in the Supplementary Material.

**Table 5:**
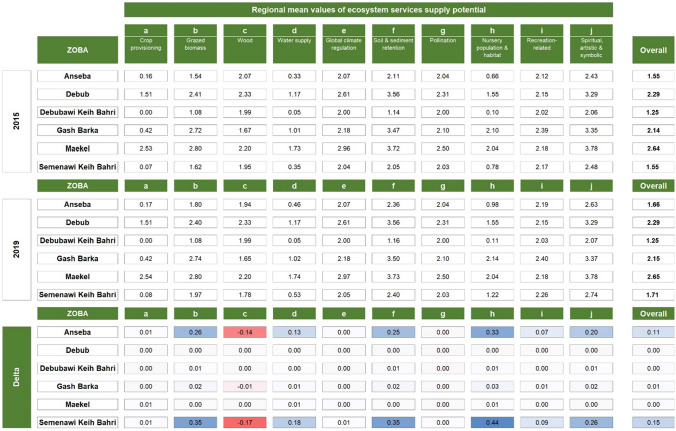
Average ES supply potential at the regional level and its changes over the period 2015 to 2019, considering the maximum values from the literature (see Table 1). For each ES, the regional mean value per unit area is reported for the years 2015 and 2019, and in terms of change (highlighted in a red to blue color scale)

**Fig. 5:**
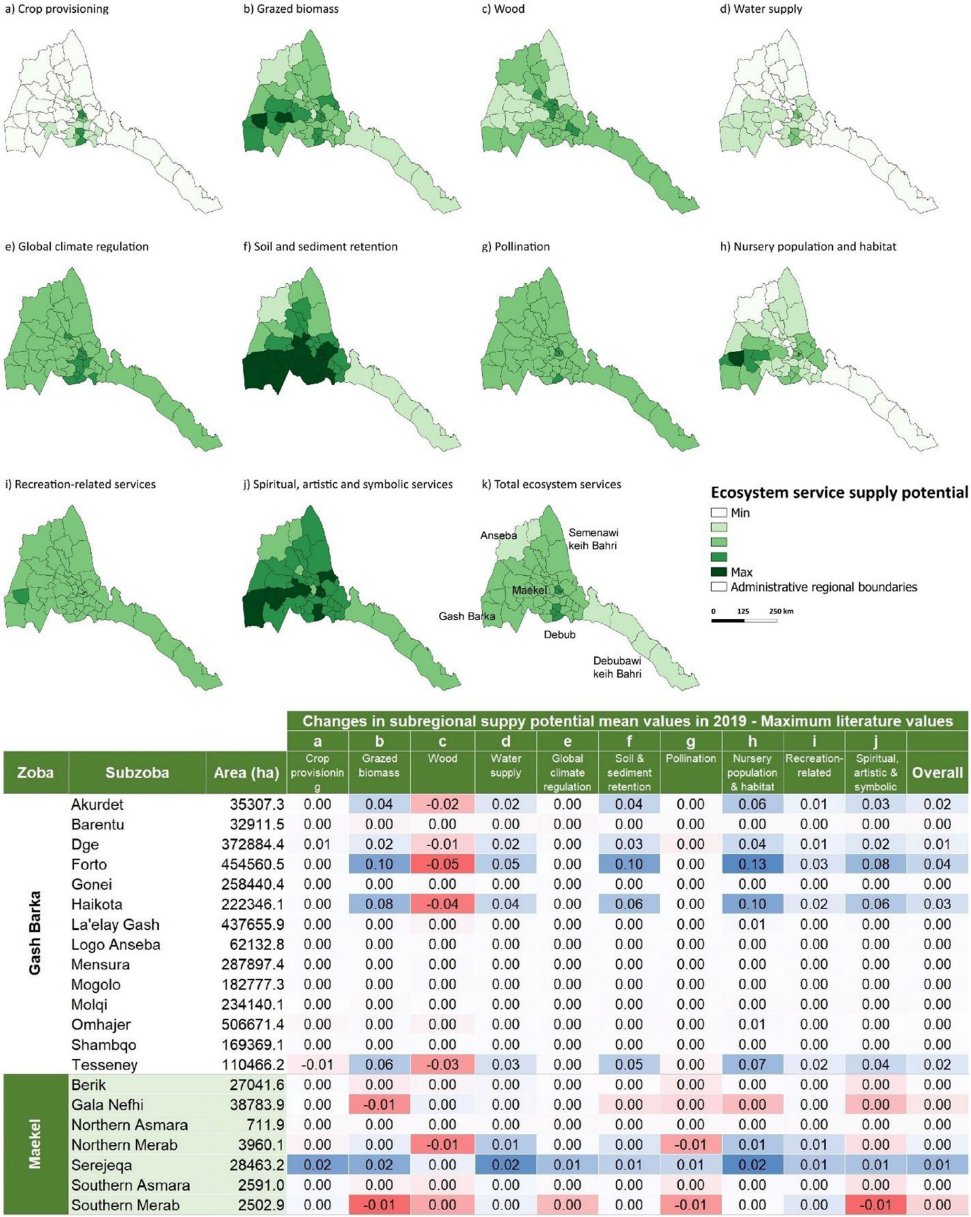
Maps of average values of ES supply potential in 2019 at the sub-regional level (top) and changes with respect to 2015 (bottom). For each ES, the maps present the mean value per unit area for the year 2019 and the table shows the mean values changes for the two illustrative regions of Gash Barka and Maekel regions (highlighted in a red to blue color scale)

The potential supply of ES also shows differences at the sub-regional level (see Fig. 5 for 2019). In general, Sel'a in Anseba region, Southern SRS in Debubawi Keih Bahri and Afabet in the Semenawi Keih Bahri region proved to be the sub-regions with the highest total supply, mainly due to their aerial extent (see Supplementary Material). Looking at the mean values, no clear pattern emerged, apart from generally higher mean values for the Maekel region versus lower mean values for the Debubawi Keih Bahri region.

Focusing on the Gash Barka and Maekel regions, the sub-regions with the highest average values per unit area are Tesseney and Hikota (Gash Barka) and Serejeqa and Northern Mereb (Maekel). On the other hand, Omhajer and La'elay Gash (Gash Barka) and Gala Nefhi and Serejeqa (Maekel) are the sub-regions with the highest total potential for ES supply. Note the generally lower total and mean values associated with the urban sub-regions (e.g., South and North Asmara).

Some considerations arise when looking at changes in the potential supply of ES compared to 2015 (Fig. 5). The sub-regions with the largest increases, both in terms of mean and total values, are Forto and Haikota in Gash Barka region and Serejeqa in Maekel region. The only sub-regions with a slight decrease in total ES potential are Barentu (Gash Barka) and Gala Nefhi and Southern Mereb (Maekel). Detailed results on the sub-regional aggregation of the potential ES supply changes compared to 2015 are included in Tables S11 and S12 in the Supplementary Material.

## Discussion

### Data and methodological approach: limitations and way forward

Our study illustrated that global land cover datasets (e.g., de Groot et al. 2012; Costanza et al. 2014) can be integrated with studies conducted under the same climate conditions to derive maximum and average values of ES. The GCLC version 3.0 shows significant advantages in mapping ES, especially for African countries, as it now includes annual change maps for the continent and the years 2015–2018 (Buchhorn et al. 2020). Compared to other land cover datasets such as Corine Land Cover and Global Land Cover (Wang et al. 2015), the GCLC version 3.0 provided by the Copernicus Land Monitoring Service stands out as one of the most suitable and beneficial for the Eritrean territory. Contrasting to most other publicly available datasets (e.g., Corine Land Cover), which include mixed land use classes such as heterogeneous agricultural land and vegetation, GCLC version 3.0 is less ambiguous, with rather limited overlaps in the depiction of ecosystems (Paprotny et al. 2021). The classification of the GCLC proved adequate to capture the primary ES of Eritrea such as water supply and crop provisioning, and its consistent and transparent nomenclature (Buchhorn et al. 2020) is worthwhile for MAES to detect the spatiotemporal changes in ES with scientific rigor (Wang et al. 2019), ensuring at the same time the transferability of our methodology to other similar contexts and nation-wide assessment.

Despite these advantages, two main limitations of the GCLC database produce some uncertainties in the interpretation of the results. The first one concerns the thematic accuracy of the maps (Tsendbazar et al. 2021), which affects especially the interpretation of land cover transitions between classes with overall small changes. Considering the classification accuracy, the latter could rather be the result of misclassifications. The analysis of the full temporal series (see Supplementary Material—Table S5) as opposed to the simple comparison of the two maps for 2015 and 2019 aimed at reducing as much as possible this potential source of uncertainty. The second limitation with potential effects on the results concerns the short temporal coverage of the analysis. Some land cover changes, including human-induced changes such as tree planting, cannot be immediately visible in remotely sensed images. This limitation is more likely to induce an underestimation of the actual changes on the ground.

The ES matrix approach allowed us to map ES supply across the Eritrean territory, using average and maximum potential values per ecosystem type reported in Table 1. Despite some identified limitations (Jacobs et al. 2015), the ES matrix-based approach is very common in MAES studies, especially at large scales and in data scarce contexts (Martínez-Harms and Balvanera 2012), where data requirements limit the application of more refined methods. The approach enables a rapid, spatially explicit assessment of ES that are linked to defined spatial units (Burkhard et al. 2014; Campagne et al. 2020). In our application, the average and maximum values assigned to each ecosystem type come from a targeted review of case studies conducted in similar climatic contexts (see Table 1). While we recognize that ES assessments are highly context-specific and data transferability is limited (Luederitz et al. 2015), we emphasize here that our assessment considers the potential ES supply, rather than the actual supply and use. The latter depends, among other things, on the institutional and governance settings that characterize the analyzed socio-ecological system, which may result in different levels of ES supply from ecosystems with similar potentials (Sieber et al. 2021). Even with this caveat, however, the assumed values may not be fully representative of the Eritrean context, as we did not include information on ecosystem conditions (Rendon et al. 2019). In this sense, a comparison of the results obtained using the average and the maximum values in the literature (e.g., in Table 4) can provide a hint on the range of variability in ES potential due to the underlying conditions. Due to limited resources, it was impossible to actively involve stakeholders in workshops in the study area (following the example by Cabral et al., (2021)) which might have led to further results even more attuned to local conditions (Roche and Campagne 2019). On the other hand, in favor of the ES matrix approach, researchers from Stanford's Natural Capital Project realized that more complex methods and models are not necessarily perceived as more salient by decision-makers, whereas they are often considered more credible by scientific experts (Ruckelshaus et al. 2015). In each application, important trade-offs in method selection need to be considered in terms of credibility, salience, and legitimacy as perceived by study audiences (Cash et al. 2003; Adem Esmail and Geneletti 2017).

In line with the idea of a tiered approach (Grêt-Regamey et al. 2015), the results presented in this paper could be coupled with the assessment of ES using more sophisticated modeling approaches or other ES indicators (cf. studies by Berta Aneseyee et al. 2020 to use the INVEST model to assess ES in Ethiopia). This would also allow to examine the extent to which the perception of relevant stakeholders may differ from each other, from literature-based values, and from those derived from different modeling approaches (Roche and Campagne 2019), and create opportunities for learning between different expert communities. Identifying areas where stakeholders' perceptions of ES provision differ significantly from each other and from the model results, so-called hard-spots, would help to prioritize further investigation (Larondelle et al. 2016).

Other important future developments relate to the integration of these results into policy and planning tools, e.g., through targeted data dissemination activities (interactive Dashboard or WebGIS), by feeding the results into nationally established environmental impact assessments and strategic environmental assessment processes (Geneletti 2013; UNEP (United Nations Environment Programme) 2014) as well as future SEEA Ecosystem Accounting for the country (SEEA-EA 2021).

The use of higher spatial data resolution should also be sought to provide meaningful results at more detailed scales of analysis. Our methodology has benefited from the spatial data consistency in thematic resolution, especially the level of classification detail of the sampling units (Lechner and Rhodes 2016), and we argue that both spatial and thematic resolution should be given special consideration in future ES assessments to offset uncertainties in patterns change recognition for policy implications. An analysis of the variability and ES assessment within lower uncertainties should be considered in future studies to improve the consistency of the results (Campagne et al. 2017, 2020). In addition, the short time series we were able to investigate, 2015–2019, makes the trend results less reliable as they could be affected by potential misclassifications. Therefore, there is a need to continue working on longer time series as data become available.

Finally, we recognize the importance of integrating the knowledge of indigenous peoples and local experts into ecosystem management. Engaging relevant stakeholders and knowledge holders from the earliest stages of the process is a key requirement (e.g., Adem Esmail and Geneletti 2017; Geneletti et al. 2020) in MAES. In this sense, our review of the empirical application of the matrix approach showed high variability in potential values associated with different ecosystems, confirming the critical importance of coupling local knowledge with estimates from the literature. This is the main limitation of the present study, which had an extremely low level of stakeholder engagement and was primarily a scientist-driven application of MAES. Nonetheless, significant efforts have been made to ensure salience for the country's most pressing policy priorities and institutional actors. The study thus can help to demonstrate the potential of MAES to inform national and sub-national policies, including key spatially explicit recommendations, and contributes significantly to much-needed capacity building. Although the proposed approach was specifically developed for the Eritrean case, it can easily be replicated in all fourteen countries of the East African sub-region and adjacent islands, including Comoros, Djibouti, Ethiopia, Kenya, and Madagascar. Using GCLC to assess ES and the methodology presented in this paper has potential value for these countries, as well as for other cases where full local or regional data for assessments are scarcely available. Ultimately, all of this should help achieve the African Union’s vision of an integrated, prosperous, and peaceful Africa by 2063 and the associated SDGs and Aichi Biodiversity Targets (IPBES 2018).

### Potential implications for policy-making in Eritrea

Our analysis of policy documents showed that several policies exist in Eritrea to which a MAES study can contribute. For example, a MAES study could directly support the aim of the National biodiversity strategy to value, conserve, restore, and wisely use biodiversity by 2040. Another opportunity is represented by the existing legislation, acknowledging the value of forest ecosystems and ensuring their conservation. Particularly relevant are the Proclamation No. 155/2006, which establishes rules for the sustainable development of forestry and wildlife resources, including the conservation of endangered and native species, and the National Agricultural Development Strategy and Policy of 2005, which addresses strategic and policy issues on how to develop agriculture without adversely impacting the environment, for example, recommending the expansion of forest enclosures and providing forest tenure rights to villages. Other contexts for MAES application are the Water Law, Proclamation No. 162/2010, and initiatives such as the 2002 “National Action Program to Combat Desertification and mitigate the effects of Drought” and the “Five Year Action Plan for The Great Green Wall Initiative (2011–2015).”

Our analysis of changes in Eritrean ecosystems showed a very high increase in absolute terms of herbaceous vegetation (+ 4465 km^2^) against a decrease in bare/sparse vegetation (− 4575 km^2^), especially in the Semenawi Keih Bahri and Anseba regions. It also highlighted the percentage increases in Herbaceous wetlands in the Maekel region (+ 429%), Permanent water bodies in the Anseba region (+ 129%) and Herbaceous vegetation in the Semenawi Keih Barhi region (+ 60%). Although it is beyond the scope of this study to identify the actual causes of the observed changes in ecosystems, some considerations can be made regarding some specific changes observed. Many of the observed changes can be associated with the construction of dams (see Box S1 in the Supplementary Material), a key action of the ambitious Soil and Water Conservation and Greening Campaign promoted by the Government of Eritrea to achieve water and food security among others. Since independence, about 785 large and small dams have been built with local resources amounting to several millions of USD. According to national authorities, around 85 to 90% of citizens living in towns and cities and 75% of citizens living in rural areas became beneficiaries of drinking water supply (MoI, 2021, last accessed on 12/12/2021).

Our analysis of potential impacts of ecosystem changes on the supply of key ES revealed a slight increase (+ 0.07%) in the overall potential supply, especially in the Semenawi Keih Bari and Anseba regions. It also revealed that the largest increases were recorded for Nursery population and habitat (+ 0.19), Soil and sediment retention (+ 0.15), and Grazed biomass (+ 0.15) against a decrease of Wood supply (-0.07). Yet, the average supply potential of Eritrean ecosystems remains low. In fact, high values of several ES are most commonly associated with closed and open forests and to a lesser extent with cultivated areas (Qiu and Turner 2013) and wetlands (de Groot et al. 2012). Therefore, in this study, the scarcity of these land cover classes drives the low national averages, and their uneven distribution explains the differences observed at the regional level, with the Debubawi Keih Bahri region generally recording lower values for multiple ES compared to higher values in the Gash Barka, Debub, and Maekel regions.

Nursery population and habitat as well as Water supply are among the services that Eritrean ecosystems have the lowest overall potential to supply, hence their increase is particularly important. Improvements in water supply go in the direction of the main objectives of the Water Law, which aims at ensuring clean, safe, and sufficient water supply for all citizens. Similarly, the increase in Soil and sediment retention is an important contribution to halting land degradation, in line with several international, national, and regional initiatives to combat desertification and its consequences. Overall, changes in ES are mostly associated with changes in land cover from bare soil to herbaceous vegetation. As a result, the most important improvements are observed in the two regions where most areas were converted, i.e., the Semenawi Keih Bahri and Anseba regions.

The above examples show how knowledge of ES can support policy implementation monitoring to ensure that changes on the ground follow the directions of objectives formulated by stakeholders and decision-makers. However, analyzing the current ES supply potential and its trend can also support the identification of locally targeted interventions to meet the needs of areas characterized by different conditions, as is the case of the maps that we provided at the regional and sub-regional levels.

## Conclusion

This study contributes an operational methodology based on open data and software that has generated new insights into the dynamics of ecosystems and their services in Eritrea. Our results can be integrated into the design of future policies with a direct impact on ES and biodiversity conservation, for example, on SEEA-EA accounts, thereby enhancing sustainable management of natural capital at national and sub-national levels. Thus, we expect this study to provide a starting point for the assessment of ecosystems and their services in Eritrea and more widely in the East African sub-region and adjacent islands.

Building on this study, the changes in ecosystems and their potential impacts of ES should be the subject of further reflection with the engagement of relevant stakeholders and even the public. An active engagement can promote a sense of shared responsibility for Eritrea’s development and conservation policies—at both national and sub-regional levels—toward co-creation and management and governance of ecosystems for sustainability.

With appropriate involvement of key stakeholders, the proposed approach can help create new spaces of knowledge sharing and co-creation—at both national and Eastern Africa sub-region levels—for the elaboration of development and conservation policies based on the concept of ecosystem services.

## Supplementary Information

Below is the link to the electronic supplementary material.

Supplementary file1 (PDF 5149 kb)
