# Supplementary material for: Mapping and assessing ecosystem services for sustainable policy and decision-making in Eritrea
Source: Ambio. 2023 Mar 18;52(6):1022–39. doi: 10.1007/s13280-023-01841-4 (PMC10024525; doi:10.1007/s13280-023-01841-4)
Supplement: Supplementary file 1 — Supplementary file1 (PDF 5149 kb) [file 13280_2023_1841_MOESM1_ESM.pdf]

Ambio

Supplementary Material (Online)
This supplementary information has not been peer reviewed.

Title: Mapping and assessing ecosystem services for sustainable policy and decision making in Eritrea.

Auhtors: Adem Esmail, B.[1]; Cortinovis, C.[2,4] Wang, J.[1,3], Geneletti, D.[4]; Albert, C.[1]

[1]. Ruhr University Bochum, Institute of Geography, Universitätsstr. 150, 44805 Bochum, Germany
[2]. Department of Geography, Humboldt-Universität zu Berlin, Germany
[3]. Department of Urban Studies and Planning, The University of Sheffield, Western Bank, Sheffield, UK
[4]. Dep. of Civil, Environmental & Mechanical Engineering, University of Trento, 38123 Trento, Italy

Abstract

The mapping and assessment of ecosystems and their services (MAES) is key to inform sustainable policy and decision-making at national and sub-national levels. Responding to the paucity of research in sub-Saharan Africa, we conduct a pilot study for Eritrea that aims to map and assess the temporal dynamics of key ecosystems and their services. We reviewed policy and legal documents, analysed land cover changes, and estimated the potential for ecosystem services supply through an expert-based matrix approach. Our results showed that from 2015 to 2019, the potential supply of the ecosystem services analysed (e.g., crop provisioning, water supply and recreation) increased, with the exception of wood supply. Overall, our study presents policy-relevant insights as to where to conserve, develop or restore ecosystem services supply in Eritrea. Our approach is transferable to similar data-scarce contexts and can thereby support policies towards more sustainable land development for people and nature.

Corresponding Author: Blal Adem Esmail (blal.ademesmail@rub.de)

Contents

Section 1 - Eritrean institutional, legal, and policy context and selected ES 3
Section 2 - Land use changes: regional and sub-regional statistics 5
National level aggregation 6
Regional aggregation 7
Sub-regional aggregation 8
Section 3 - ES mapping and assessment 9
National aggregation 12
Regional aggregation 13
Sub-regional aggregation 14
Section 4 - Links with ongoing national initiatives 16

Section 1 - Eritrean institutional, legal, and policy context and selected ES

Table S 1. Six administrative regions and 57 sub-regional administrative districts in Eritrea pursuant to Proclamation No. 86/1996. Focus on the Maeke (Central) region, where the capital Asmara is located, and Gash Barka Region, considered the breadbasket and a biodiversity hotspot.

Maekel	Anseba	Debub	Gash Barka	Semenawi Keih Bahri	Debubawi Keih Bahri
Southern Merab	Keren	Mendefera	Omhajer	Massawa	Assab
Southern Asmara	Geleb	Dbarwa	Dge	Sheb	Southern SRS
Gala Nefhi	Hagaz	Segeneiti	Akurdet	Afabet	Central SRS
Northern Asmara	Adi Tekeliezan	Dekemhare	Mensura	Quarura	Ara'eta
Serejeqa	Elabered	Mai Mne	Forto	Gel'alo	
Northern Merab	Habero	Adi Kuala	Molqi	Nakfa	
Berik	Asmat	Tsorona	Mogolo	Adobhe	
	Sel'a	Senafe	Gonei	Foro	
	Hamelmalo	Adi Keih	Logo Anseba	Gindae	
	Halhal	Areza	Tesseney		
	Kerkebet	Emni Haili	Haikota		
		Mai Aini	La'elay Gash		
			Barentu		
			Shambqo		

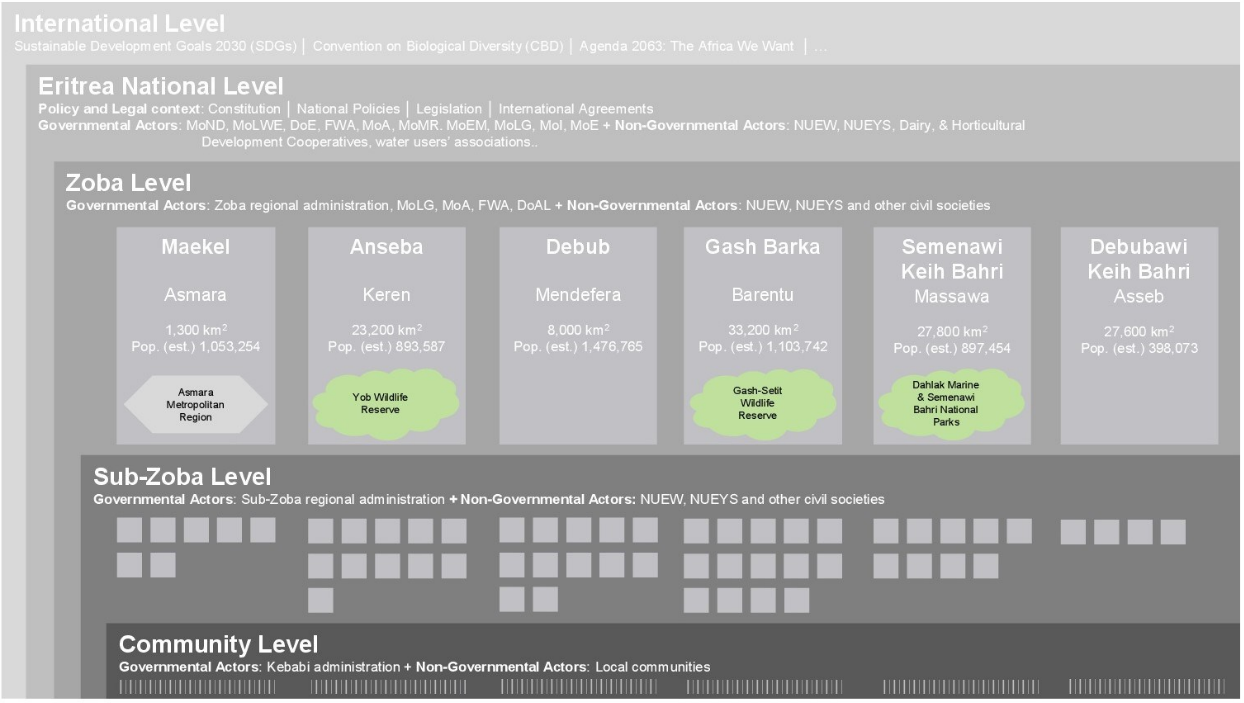

Figure S 1. The four-tiered system of governance in Eritrea. Highlight on the metropolitan region of Asmara and three main protected areas, and population distribution at the community level.

Table S 2. List of relevant policy and legal documents in Eritrea based on of relevance for biodiversity and MAES applications. AGR -Agricultural and rural development, CP - Cultivated plants, ENV – Environment, FISH – Fisheries, FOR – Forestry, L&S -Land and soil, SEA – Sea, SOP- Social protection, WAT – Water, WES - Wild species and ecosystems, MSC - Miscellaneous (Classification according to FAOLex Database, http://www.fao.org/faolex, last accessed 23/07/2021).

Code	Title	Year	FAO Classification
CS1	Constitution	1997	
**Code**	**National Policy Documents**	**Year**	**FAO Classification**
NP1	Nationally Determined Contributions (NDCs) Report to UNFCCC 2018.	2018	MSC
NP2	Revised National Biodiversity Strategy and Action Plan for Eritrea (2014-2020).	2014	ENV
NP3	Health Sector Strategic Development Plan 2012-2016	2011	n.a.
NP4	National Health Policy	2010	SOP
NP5	Integrated Water Resource Management Action Plan	2009	WAT
NP6	The Five Year Indicative Development Plan (FYIDP), GOE	2009	ENV
NP7	Ten Year Long-Term Indicative Perspective Development Plan (TYIPDP), GOE	2009	n.a.
NP8	National Adaptation Programme of Action (NAPA)	2007	ENV
NP9	Poverty Reduction Strategy Paper (PRSP)	2004	MSC
NP10	Interim-Poverty Reduction Strategy Paper (I-PRSP), GOE	2003	MSC
NP11	National Economic Policy Framework and Program (NEPFP), GOE	1998-2000	n.a.
NP12	Proclamation for the Establishment of Local Governments No. 86	1996	MSC
NP13	Macro Policy, GOE	1994	L&S
NP14	National Charter	1994	L&S
	**Biodiversity**	**Year**	
BD1	Forest and Wildlife Conservation and Development Proclamation No. 155/2006	2006	MSC
BD2	National Action Plan for the Conservation of Marine Turtles and their Habitats (Draft)	2006	SEA
BD3	Forest and Wildlife Policy	2005	n.a.
BD4	Proclamation on Conservation of Biodiversity	1998	n.a.
	**Land Degradation**	**Year**	
LD1	Five Year Action Plan for The Great Green Wall Initiative (2011-2015) (Draft)	2011	L&S
LD2	Land Use Policy	2007	AGR
LD3	National Action Program to Combat Desertification and mitigate the effects of Drought (NAP, 2002)	2002	L&S
LD4	Land Use Planning Regulatory Framework	1999	n.a.
LD5	Land Proclamation No.58	1994	L&S
	**Climate Change and Energy**	**Year**	
CC1	Second National Communication (SNC)	2012	ENV
CC2	Renewable Energy Sub-Sector Policy	1997	AGR
	**Cross-cutting: Environment**	**Year**	
EN1	Environmental Protection and Management Regulations, Legal Notice 127/20017	2017	ENV
EN2	Eritrean Environmental, Protection Management, and Rehabilitation Framework 179/20017	2017	ENV
EN3	Environmental law Proclamation (Draft)	2012	ENV
EN4	Regulation for the issuance of permit for the importation or exportation of ODS and ODS based equipment or products (legal notice No. 11	2010	MSC
EN5	National Bio-safety Framework (NBF)	2007	MSC
EN6	Legal notice for banning of plastic bags No. 99	2004	MSC
EN7	Environmental Health Policy	1998	n.a.
EN8	National Environmental Assessment Procedures & Guidelines	1999	ENV
EN9	National Environmental Management Plan (NEMP)	1995	ENV
	**Cross-cutting: Marine and Fisheries**	**Year**	
MF1	Proclamation to Establish an Integrated Coastal Area Management (ICAM) (Draft)	2007	ENV
MF2	Proclamation to Establish the Eritrean Coastal Authority (Draft)	2007	L&S
MF3	Fishery Proclamation 195/2007	2007	n.a.
MF4	National Coastal Policy (Draft)	2006	L&S
MF5	Fisheries Product Proclamation	1998	FISH
	**Cross-cutting: Water**	**Year**	
WT1	Water Law, Proclamation No. 162,	2010	WAT
WT2	Integrated Water Resource Management Action Plan	2009	WAT
WT3	Water Policy	2007	WAT
WT4	Rural Sanitation Policy	2007	n.a.
	**Cross-cutting: Agricultural**	**Year**	
AG1	Proclamation for Pesticide Handling (Draft)	2006	MSC
AG2	Agriculture Sector Policy	2006	n.a.
AG3	National Agricultural Development Strategy and Policy	2005	AGR
	**Cross-cutting: Mining**	**Year**	
MN1	Proclamation to Promote the Development of Mineral Resources No. 68	1995	MSC
MN2	Regulations on Petroleum Operations Legal Notice No. 24	1995	MSC
	**Cross-cutting: Tourism**	**Year**	
TR1	Tourism Development Policy and Strategy	1999	MSC
TR2	National Tourism Development Plan (2000-2020)	1999	MSC
	**Cross-cutting: other**	**Year**	
OT1	Piloting Integrated Processes and Approaches to Facilitate National Reporting (FNR) to Rio-Conventions	2012	MSC
OT2	National Situational Analysis and Needs Assessment (SANA)	2011	SOP
OT3	National Capacity Needs Self Assessment (NCSA)	2007	MSC
OT4	Press Proclamation No 90	1996	MSC

Table S 3. Definition of pre-selected ES according to the SEEA-EA classification

ES	Definition
**a) Crop**	Ecosystem contributions to the growth of cultivated plants that are harvested for various uses including food and fibre production, fodder and energy.
**b) Grazed biomass**	the ecosystem contributions to the growth of grazed biomass that is an input to the growth of cultivated livestock (excluding the growth of crops used to produce fodder for livestock)
**c) Wood**	ecosystem contributions to the growth of trees and other woody biomass, both cultivated and uncultivated, that are harvested for various uses including timber production and energy..
**d) Water supply**	combined ecosystem contributions of water flow regulation, water purification, and other ES to the supply of water of appropriate quality to users for various uses including household consumption.
**e) Global climate regulation**	Global climate regulation services are the ecosystem contributions to the regulation of the chemical composition of the atmosphere and oceans that affect global climate through the accumulation and retention of carbon and other GHG (e.g., methane) in ecosystems and the ability of ecosystems to remove carbon from the atmosphere. This is a final ES.
**f) Soil erosion control services**	Soil erosion control services are the ecosystem contributions, particularly the stabilising effects of vegetation, that reduce the loss of soil (and sediment) and support use of the environment (e.g., agricultural activity, water supply). This is may be recorded as a final or intermediate service.
**g) Pollination**	Pollination services are the ecosystem contributions by wild pollinators to the fertilization of crops that maintains or increases the abundance and/or diversity of other species that economic units use or enjoy. This may be recorded as a final or intermediate service.
**h) Nursery population and habitat**	Nursery population and habitat maintenance services are the ecosystem contributions necessary for sustaining populations of species that economic units ultimately use or enjoy either through the maintenance of habitats (e.g., for nurseries or migration) or the protection of natural gene pools. This service is an intermediate service and may input to a number of different final ES including biomass provision and recreation-related services.
**i) Recreation-related**	Recreation-related services are the ecosystem contributions, in particular through the biophysical characteristics and qualities of ecosystems, that enable people to use and enjoy the environment through direct, in-situ, physical and experiential interactions with the environment. This includes services to both locals and non-locals (i.e. visitors and tourists). Recreation-related services may also be supplied to those undertaking recreational fishing and hunting. This is a final ES.
**j) Spiritual, artistic and symbolic**	Spiritual artistic and symbolic services are the ecosystem contributions, in particular through the biophysical characteristics and qualities of ecosystems, that are recognized by people for their cultural, historical, aesthetic, sacred or religious significance. These services may underpin people's cultural identity and may inspire people to express themselves through various artistic media. This is a final ES.

Section 2 - Land use changes: regional and sub-regional statistics

Table S 4. Spatial data for the analysis of land cover change

Name	Description	Source or reference
**Land cover 2015-base**	Baseline landcover updated: a discrete classification with 11 classes (out of 23) present in the case study, following UN-FAO's Land Cover Classification System (LCCS)	10.5281/zenodo.3939038
**Land cover 2016-conso**	Consolidated landcover for the year 2016	10.5281/zenodo.3518026
**Land cover 2017-conso**	Consolidated landcover for the year 2017	10.5281/zenodo.3518036
**Land cover 2018-conso**	Consolidated landcover for the year 2018	10.5281/zenodo.3518038
**Land cover 2019-nrt**	Near-Real-Time landcover for the year 2019	10.5281/zenodo.3939050
**Boundaries of Eritrea - AFRICOVER**	The national and administrative boundaries provided by the National Focal Pont. Coastlines adapted to match the landcover dataset. The boundaries included in the Multipurpose Africover Database for the Environmental Resources (MADE) for orientation purposes	FAO Map Catalog (last accessed 23/07/2021)

National level aggregation

Table S 5. National level land cover class distribution and their changes during the period 2016-2019 with respect to the baseline year 2015. Emphasized the subregions with the highest gains (blue) and losses (red).

Nationa land cover distribution during 2015-2019 (km^2)

YEAR	20 Shrubs	30 Herbaceous vegetation	40 Cultivated & managed vegetation/ agriculture	50 Urban / built up	60 Bare/ sparse vegetation	80 Permanent water bodies	90 Herbaceous wetland	114 Closed forest, deciduous broad leaf	116 Closed forest, unknown	124 Open forest, deciduous broad leaf	126 Open forest, unknown
2015	32104.2	21149.1	6858.9	91.8	61021.6	45.7	13.5	4.4	11.3	19.8	695.0
2016	32193.1	21111.6	6846.5	92.3	60946.9	53.3	20.9	4.3	11.2	20.0	715.0
2017	32215.3	21107.4	6836.2	93.0	60915.6	75.8	17.2	4.3	11.2	20.6	718.8
2018	32128.1	24699.7	6877.1	93.5	57345.9	76.2	22.8	4.3	11.0	21.7	734.8
2019	32055.5	25615.3	6918.4	93.6	56446.4	78.6	25.3	4.3	11.0	21.5	745.3

Nationa land cover change during 2015-2019 (km^2)

YEAR	20	30	40	50	60	80	90	114	116	124	126
2015	-	-	-	-	-	-	-	-	-	-	-
2016	88.9	-37.5	-12.4	0.6	-74.7	7.6	7.5	-0.1	0.0	0.2	19.9
2017	111.0	-41.7	-22.7	1.2	-106.0	30.1	3.7	-0.1	-0.1	0.8	23.7
2018	23.9	3550.6	18.2	1.7	-3675.7	30.5	9.3	0.0	-0.2	1.9	39.8
2019	-48.7	4466.2	59.5	1.8	-4575.2	32.9	11.8	0.0	-0.2	1.7	50.3

Nationa land cover change during 2015-2019 (%)

YEAR	20	30	40	50	60	80	90	114	116	124	126
2015	-	-	-	-	-	-	-	-	-	-	-
2016	0.3%	-0.2%	-0.2%	0.6%	-0.1%	16.7%	55.6%	-2.2%	-0.4%	1.2%	2.9%
2017	0.3%	-0.2%	-0.3%	1.3%	-0.2%	65.9%	27.6%	-2.5%	-0.8%	3.9%	3.4%
2018	0.1%	16.8%	0.3%	1.8%	-6.0%	66.7%	69.1%	-0.5%	-1.8%	9.7%	5.7%
2019	-0.2%	21.1%	0.9%	2.0%	-7.5%	72.0%	87.8%	-1.1%	-2.1%	8.5%	7.2%

Regional aggregation

Table S 6. Highlight of regional level land cover class changes for 2019 with respect to the baseline year 2015. Emphasized the subregions with the highest gains (blue) and losses (red).

Regional land cover change in 2019 with respect to 2015 (km^2)

YEAR	ZOBA	20 Shrubs	30 Herbaceous vegetation	40 Cultivated & managed vegetation/ agriculture	50 Urban / built up	60 Bare/ sparse vegetation	80 Permanent water bodies	90 Herbaceous wetland	114 Closed forest, deciduous broad leaf	116 Closed forest, unknown	124 Open forest, deciduous broad leaf	126 Open forest, unknown
2019	Anseba	-149.82	1340.43	24.10	0.36	-1228.11	7.84	3.03	0.01	-0.30	0.97	1.49
	Debub	4.66	-4.40	0.35	0.35	-5.83	2.48	0.44	0.00	0.00	0.00	1.95
	Debubawi Keih Bahri	70.30	31.33	1.35	0.20	-111.77	8.23	0.24	0.00	0.00	0.00	0.12
	Gash Barka	-30.15	194.65	3.13	0.26	-188.35	11.14	5.40	0.00	0.00	-0.16	4.07
	Maekel	-1.39	-0.85	0.14	0.27	0.00	0.21	0.72	0.00	0.00	0.00	0.88
	Semenawi Keih Bahri	57.70	2905.06	30.43	0.35	-3041.18	3.01	1.98	-0.06	0.06	0.87	41.78

Regional land cover change in 2019 with respect to 2015 (%)

YEAR	ZOBA	20	30	40	50	60	80	90	114	116	124	126
2019	Anseba	-2.5%	58.4%	3.8%	4.0%	-10.2%	129.4%	51.1%	4.8%	-28.7%	21.0%	2.1%
	Debub	0.1%	-0.4%	0.0%	1.6%	-24.8%	83.5%	127.6%	-	-	-	3.2%
	Debubawi Keih Bahri	10.2%	7.1%	10.3%	5.1%	-0.5%	56.5%	64.5%	-	-	-	1.4%
	Gash Barka	-0.2%	1.6%	0.1%	3.6%	-3.2%	71.4%	325.2%	-	0.0%	-2.7%	1.4%
	Maekel	-0.4%	-1.1%	0.0%	0.7%	0.0%	33.3%	428.6%	-	-	-	4.2%
	Semenawi Keih Bahri	0.8%	60.4%	10.6%	4.0%	-14.2%	51.4%	39.8%	-1.5%	0.6%	9.2%	17.3%

Sub-regional aggregation

Table S 7. Sub-regional level land cover class changes in the year 2019 with respect to the baseline year 2015, in percentage. Emphasized the subregions with the highest gains (blue) and losses (red).

Subregional land cover change between 2015 and 2019 (%)

ZOBA	SUBZOBA	20 Shrubs	30 Herbaceous vegetation	40 Cultivated & managed vegetation/ agriculture	50 Urban / built up	60 Bare/ sparse vegetation	80 Permanent water bodies	90 Herbaceous wetland	114 Closed forest, deciduous broad leaf	116 Closed forest, unknown	124 Open forest, deciduous broad leaf	126 Open forest, unknown
Anseba	Adi Tekeliezan	0.0%	0.0%	0.0%	1.3%	-	-	-	-	-	-	0.0%
	Asmat	-7.2%	45.8%	20.0%	75.0%	-9.3%	-	-	-	14.3%	0.0%	3.7%
	Elabered	0.0%	0.0%	0.0%	1.6%	0.0%	-	100.0%	-	-	-	0.0%
	Geleb	0.0%	0.0%	0.0%	0.0%	0.0%	-	-	-	0.0%	-	0.0%
	Habero	-4.1%	17.9%	0.6%	0.0%	-9.8%	-	-	-	-10.0%	-	2.3%
	Hagaz	-0.1%	0.2%	0.0%	6.7%	-0.1%	-	-	-	-	0.0%	0.0%
	Halhal	-2.4%	28.0%	9.5%	0.0%	0.0%	-	-	-	-	-	0.0%
	Hamelmalo	0.0%	0.0%	0.0%	50.0%	-	-	-	-	-	-	0.0%
	Keren	0.0%	-3.0%	0.2%	3.8%	0.0%	-	-	-	-	-	0.0%
	Kerkebet	-0.4%	16.7%	44.8%	0.0%	-6.6%	128.8%	-70.0%	-	-33.3%	0.0%	3.2%
	Sel'a	2.0%	260.1%	68.6%	0.0%	-11.2%	-	66.1%	4.8%	-78.8%	21.2%	3.0%
Debub	Adi Keih	0.1%	1.6%	0.1%	30.2%	-48.5%	-	-	-	-	-	0.7%
	Adi Kuala	-0.1%	-0.2%	0.0%	0.4%	0.0%	10.5%	-	-	-	-	0.0%
	Areza	0.0%	0.0%	0.0%	0.0%	0.0%	-	-	-	-	-	0.0%
	Dbarwa	0.0%	-2.1%	-0.1%	0.9%	-7.7%	133.8%	633.3%	-	-	-	0.0%
	Dekemhare	0.0%	-0.4%	0.0%	0.3%	0.0%	0.0%	200.0%	-	-	-	0.0%
	Emni Haili	0.0%	0.0%	0.0%	0.0%	0.0%	6.3%	-	-	-	-	0.0%
	Mai Aini	0.0%	0.0%	0.0%	0.0%	0.0%	0.0%	0.0%	-	-	-	0.0%
	Mai Mne	0.0%	0.0%	0.0%	0.0%	-	-	-	-	-	-	0.0%
	Mendefera	0.0%	-0.2%	0.0%	0.7%	0.0%	3.6%	50.0%	-	-	-	0.0%
	Segeneiti	0.0%	-0.1%	0.0%	0.0%	0.0%	20.0%	-	-	-	-	0.0%
	Senafe	0.5%	-1.5%	0.4%	2.3%	-46.2%	10.7%	-	-	-	-	8.2%
	Tsorona	0.0%	0.0%	0.0%	0.0%	0.0%	-	-	-	-	-	0.0%
Debubawi Keih Bahri	Ara'eta	15.5%	17.1%	12.9%	0.0%	-1.5%	51.3%	69.2%	-	-	-	0.4%
	Assab	52.2%	-8.7%	113.0%	0.7%	-0.5%	-8.3%	-	-	-	-	-
	Central SRS	1.0%	0.0%	9.1%	-	0.0%	11.2%	-	-	-	-	-
	Southern SRS	4.4%	-1.3%	2.7%	250.0%	-0.1%	73.6%	40.0%	-	-	-	1.3%
Gash Barka	Akurdet	-0.3%	3.0%	0.6%	4.9%	-3.1%	-	-	-	0.0%	-	0.7%
	Barentu	0.0%	-0.1%	0.0%	19.8%	0.0%	-	-	-	-	-	-
	Dge	-0.3%	1.1%	6.7%	0.0%	-1.7%	164.8%	28.9%	-	5.3%	-	1.4%
	Forto	-0.5%	9.4%	1.3%	0.0%	-3.7%	88.4%	-	-	0.0%	-33.3%	44.7%
	Gonei	-0.1%	0.1%	0.1%	0.0%	-1.8%	-	-	-	-	0.0%	0.0%
	Haikota	-3.2%	2.9%	-0.2%	0.0%	-12.4%	-0.8%	680.0%	-	-	-31.4%	3.8%
	La'elay Gash	-0.2%	0.2%	0.4%	0.0%	0.0%	-	1000.0%	-	-100.0%	0.0%	-0.1%
	Logo Anseba	0.0%	0.0%	0.0%	0.0%	0.0%	-	200.0%	-	-	-	0.0%
	Mensura	0.0%	0.0%	0.0%	0.0%	-0.1%	-	-	-	-	-	5.5%
	Mogolo	0.0%	0.0%	0.1%	-	-0.1%	-	-	-	-	-	0.0%
	Molqi	0.0%	-0.2%	0.3%	0.0%	0.0%	-	-	-	0.0%	0.0%	0.0%
	Omhajer	-0.1%	0.3%	-0.5%	0.0%	-7.1%	-11.2%	910.0%	-	-	0.0%	0.7%
	Shambqo	0.0%	0.0%	0.0%	5.0%	0.0%	-	200.0%	-	-	0.0%	-0.1%
	Tesseney	-1.0%	3.5%	-1.1%	0.0%	-6.8%	17.4%	3033.3%	-	-	0.0%	0.6%
Maekel	Berik	-0.1%	-0.6%	0.0%	0.8%	0.0%	150.0%	450.0%	-	-	-	0.0%
	Gala Nefhi	0.2%	-1.8%	0.0%	0.9%	0.0%	90.9%	-	-	-	-	0.0%
	Northern Asmara	0.0%	0.0%	0.0%	0.0%	-	-	-	-	-	-	0.0%
	Northern Merab	-1.6%	-0.3%	-0.2%	0.5%	-	33.3%	140.0%	-	-	-	0.0%
	Serejeqa	-1.3%	0.7%	0.2%	2.8%	-	-29.2%	625.0%	-	-	-	5.1%
	Southern Asmara	-0.2%	-0.5%	0.0%	0.1%	-	-	-	-	-	-	0.0%
	Southern Merab	-2.1%	-1.3%	-0.2%	0.9%	-	-	133.3%	-	-	-	0.0%
Semenawi Keih Bahri	Adobhe	0.9%	22.9%	-1.9%	0.0%	-2.8%	-	88.9%	-	-	-25.0%	-7.7%
	Afabet	2.2%	102.3%	31.4%	0.0%	-21.0%	14.7%	19.2%	-	-	-	0.0%
	Foro	0.8%	5.3%	-2.5%	0.0%	-7.3%	335.0%	-	-	-	-	-1.6%
	Gel'alo	1.8%	2.5%	-9.9%	0.0%	-0.4%	38.5%	63.6%	-	-	-	2.2%
	Gindae	0.4%	0.3%	1.4%	3.4%	-14.0%	-	-	-2.0%	0.7%	2.9%	30.4%
	Massawa	93.1%	1.9%	-27.2%	6.4%	-2.3%	173.3%	50.0%	-	-	-	128.6%
	Nakfa	-4.6%	96.2%	3.9%	0.0%	-22.1%	-	-	-	-	0.0%	-2.9%
	Quarura	13.4%	126.8%	329.4%	-	-21.9%	7.1%	93.3%	-	-	140.9%	23.3%
	Sheb	0.6%	118.7%	39.6%	0.0%	-39.5%	100.0%	-	0.0%	0.0%	-0.3%	2.4%

Section 3 - ES mapping and assessment

*Table S 8. Studies included as evidence for our methodology for assessing potential ES supply in Step 3. * studies not considered in the statistics*

Authors	Title	Country	Scale	N. of Ecosystems	ES classification	No. of ES			ES prioritatization	Scoring scale	Involved actors
						Provisioning	Regulating	Cultural			Involved actors
Kilonzi and Ota, (2019)	Ecosystem service preferences across multilevel stakeholders in co-managed forests: Case of Aberdare protected forest ecosystem in Kenya	Kenya	Regional	1	MA	7	6	3	-	Likert scale: 0-5	15 multilevel stakeholders/organization
Malherbe et al., (2019)	Mapping the Loss of Ecosystem Services in a Region Under Intensive Land Use Along the Southern Coast of South Africa	South Africa	Regional	11	n.a.	0	5	4	-	Likert scale: 0-5	
Owuor et al., (2017)	Mapping of ecosystem services flow in Mida Creek, Kenya	Kenya	Regional	8	MA	5	4	4	-	Likert scale: 0-5	Community representatives and park and reserve managers
Sanchez-Porras et al., (2018)	Evaluation of the Potential Change to the Ecosystem Service Provision Due to Industrialization	Mexico	Regional	13	MA	10	11	5	-	Likert scale: 0-5	Experts (?)
Sun et al., (2020)	Spatiotemporal patterns and drivers of ecosystem service supply and demand across the conterminous United States: A multiscale analysis	USA	Continental	11	n.a.	7	8	3	-	Likert scale: 0-5	10 professors and researchers
Vrebos et al., (2015)	Mapping ecosystem service flows with land cover scoring maps for data-scarce regions	Uganda	Regional	15	TEEB	5	7	2	-	Likert scale: 0-5	Team of local researchers
Wangai et al., (2019)	Quantifying and mapping land use changes and regulating ecosystem service potentials in a data-scarce peri-urban region in Kenya	Kenya	Regional	6	CICES	0	7	0	Importance 1-10	Likert scale: 0-5	113 local respondents orally interviewed + 11 experts
Wangai et al., (2017)	Contributing to the cultural ecosystem services and human wellbeing debate: a case study application on indicators and linkages	Kenya	Regional	n.a.	CICES	0	0	3	Importance 1-11	Likert scale: 0-5	113 local respondents + 11 experts
Martínez-Harms et al., (2016)*	Enhancing ecosystem services maps combining field and environmental data	Mexico	Regional	8	n.a.	3	0	0	-	Actual quantities	None
Sinare et al., (2016)*	Assessment of ecosystem services and benefits in village landscapes - A case study from Burkina Faso	Burkina Faso	Local	8	CICES	5	0	0	-	Actual quantities	Villagers

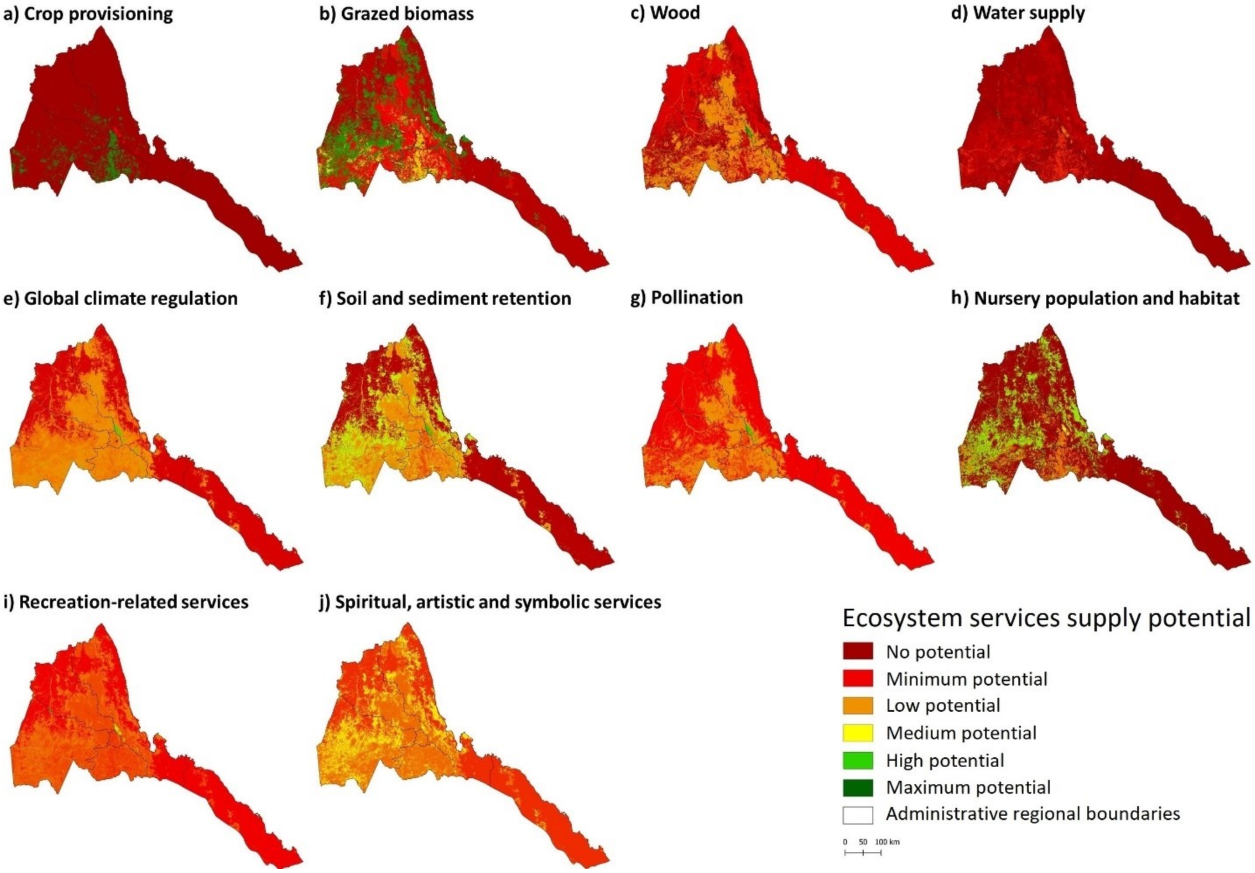

Figure S 2. ES supply potential in Eritrea based on the average literature value.

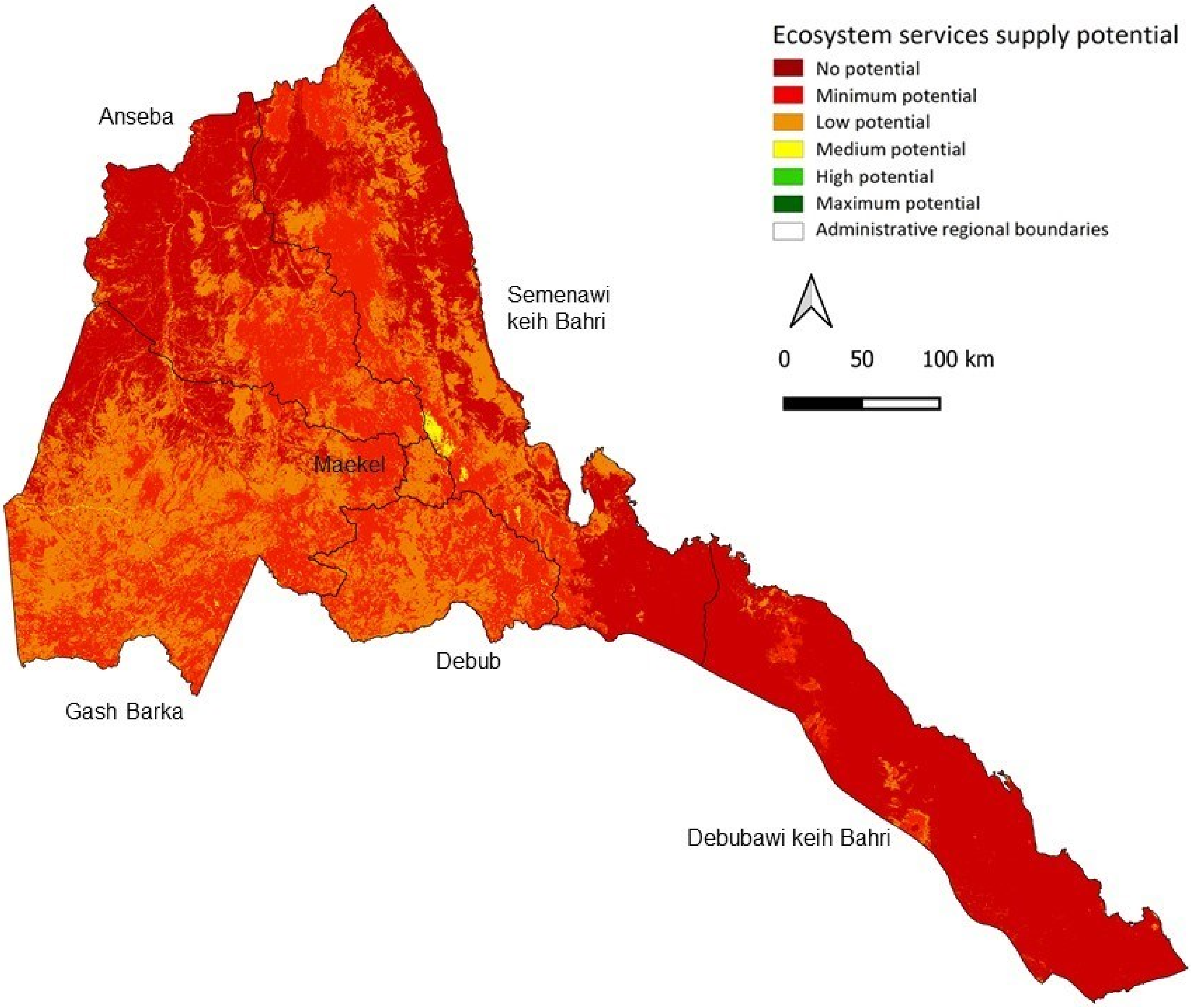

Figure S 3. Overall ES potential map based on the average values

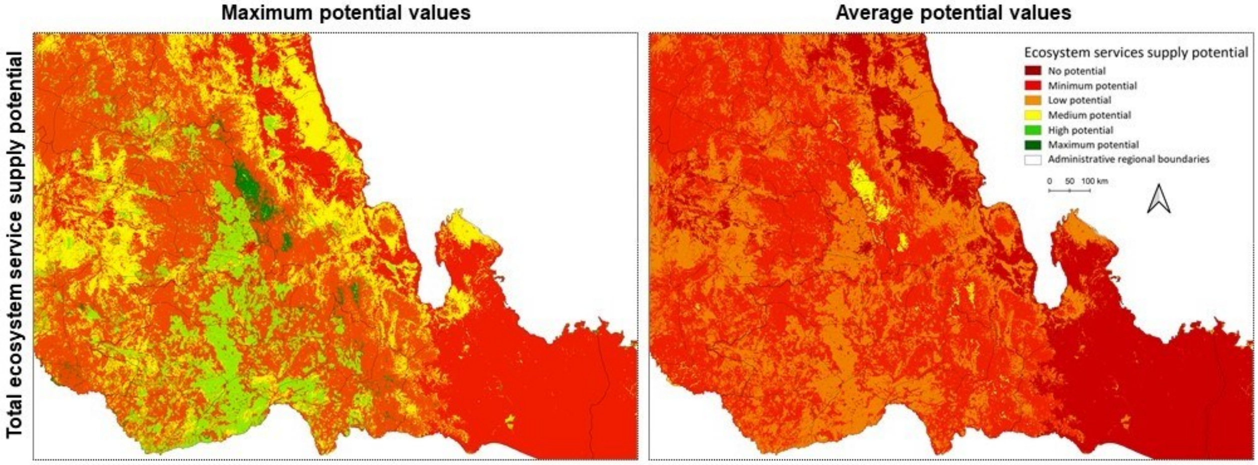

Figure S 4. Overall ES potential maps: comparison between maximum values (left) and average values (right).

National aggregation

Table S 9. National ES supply potential and its changes during the period 2015 to 2019, considering both maximum and average literature values. For each ES the national mean value per unit area (top), and total values are reported for the years 2015 and 2019, and in terms of difference. Emphasized the services with the highest gains (blue) and losses (red).

		National mean values of ecosystem services supply potential										
		a Crop provisioning	b Grazed biomass	c Wood	d Water supply	e Global climate regulation	f Soil & sediment retention	g Pollination	h Nursery population & habitat	i Recreation-related	j Spiritual, artistic & symbolic	Overall
Maximum	2015	0.31	1.88	1.93	0.55	2.13	2.42	2.07	1.07	2.19	2.71	**1.73**
	2019	0.31	2.03	1.86	0.63	2.13	2.57	2.07	1.26	2.23	2.82	**1.79**
	Delta	0.00	0.15	-0.07	0.08	0.00	0.15	0.00	0.19	0.04	0.11	**0.07**
Average	2015	0.26	1.35	0.99	0.17	1.31	1.36	1.31	0.73	1.30	1.70	**1.05**
	2019	0.27	1.51	0.96	0.19	1.36	1.47	1.31	0.86	1.33	1.76	**1.10**
	Delta	0.00	0.16	-0.03	0.02	0.04	0.11	0.00	0.13	0.03	0.06	**0.05**

		National total ecosystem services supply potential										
		a	b	c	d	e	f	g	h	i	j	Overall
Maximum	2015	3,118,203	19,197,927	19,737,418	5,594,776	21,750,472	24,684,839	21,148,396	10,922,298	22,407,190	27,664,557	**17,622,608**
	2019	3,162,403	20,722,655	18,991,650	6,397,961	21,775,098	26,220,121	21,157,979	12,847,594	22,804,507	28,822,626	**18,290,260**
	Delta	44,200	1,524,728	-745,768	803,185	24,626	1,535,282	9,584	1,925,296	397,317	1,158,069	**667,652**
Average	2015	2,703,902	13,795,449	10,089,476	1,764,157	13,418,588	13,882,709	13,372,787	7,446,139	13,307,232	17,401,041	**10,718,148**
	2019	2,732,136	15,382,879	9,798,163	1,981,843	13,846,648	15,025,625	13,381,787	8,793,679	13,626,925	17,974,574	**11,254,426**
	Delta	28,234	1,587,431	-291,312	217,686	428,060	1,142,917	9,000	1,347,540	319,693	573,533	**536,278**

Regional aggregation

Table S 10. Regional ES supply potential and their changes, based on maximum literature values. For each ES the regional total values are reported for the years 2015 and 2019, and in terms of difference. Emphasized the services with the highest gains (blue) and losses (red).

		Regional mean values of ecosystem services supply potential										
	ZOBA	a Crop provisioning	b Grazed biomass	c Wood	d Water supply	e Global climate regulation	f Soil & sediment retention	g Pollination	h Nursery population & habitat	i Recreation-related	j Spiritual, artistic & symbolic	Overall
2015	Anseba	288,707.00	2,704,216.00	3,643,264.00	578,483.00	3,639,966.00	3,706,403.00	3,583,194.20	1,156,574.00	3,730,957.00	4,271,367.80	2730313.27
2015	Debub	1,220,510.00	1,944,537.00	1,884,949.00	948,204.00	2,108,480.00	2,876,446.00	1,867,683.80	1,254,004.00	1,735,403.00	2,655,755.40	1849485.83
2015	Debubawi Keih Bahri	8359.00	2057859.00	3795214.00	86850.00	3816297.00	2180853.00	3812718.80	198203.00	3855650.00	3932474.40	2374447.91
2015	Gash Barka	1,170,277.00	7,660,880.00	4,697,527.00	2,837,242.00	6,141,985.00	9,792,958.00	5,915,614.60	5,932,419.00	6,751,267.00	9,454,933.60	6035510.45
2015	Maekel	220,501.00	243,823.00	191,417.00	150,593.00	257,986.00	324,307.00	217,867.20	177,564.00	189,744.00	329,154.00	230295.62
2015	Semenawi Keih Bahri	209849.00	4586612.00	5525047.00	993404.00	5785758.00	5804986.00	5751317.00	2203534.00	6144169.00	7020871.40	4402554.87
	ZOBA	a	b	c	d	e	f	g	h	i	j	Overall
2019	Anseba	300,033.00	3,160,058.00	3,405,184.00	814,453.00	3,644,996.00	4,140,739.00	3,584,089.40	1,729,288.00	3,846,237.00	4,616,631.80	2924170.99
2019	Debub	1,221,381.00	1,943,552.00	1,886,053.00	949,595.00	2,109,043.00	2,876,446.00	1,867,654.60	1,254,349.00	1,736,251.00	2,655,863.00	1850018.77
2019	Debubawi Keih Bahri	9004.00	2068094.00	3794452.00	96030.00	3816559.00	2206383.00	3811442.80	215286.00	3860427.00	3942786.20	2382046.49
2019	Gash Barka	1,173,803.00	7,728,691.00	4,661,031.00	2,879,187.00	6,144,353.00	9,860,802.00	5,914,389.40	6,023,299.00	6,772,273.00	9,508,402.40	6066623.21
2019	Maekel	220,977.00	243,996.00	191,523.00	151,247.00	258,306.00	324,504.00	217,952.60	178,051.00	190,092.00	329,262.20	230591.08
2019	Semenawi Keih Bahri	237205.00	5578264.00	5053407.00	1507449.00	5801841.00	6811247.00	5762450.60	3447321.00	6399227.00	7769680.00	4836809.30
	ZOBA	a	b	c	d	e	f	g	h	i	j	Overall
Delta	Anseba	11,326.00	455,842.00	-238,080.00	235,970.00	5,030.00	434,336.00	895.20	572,714.00	115,280.00	345,264.00	193857.73
Delta	Debub	871.00	-985.00	1,104.00	1,391.00	563.00	0.00	-29.20	345.00	848.00	107.60	532.94
Delta	Debubawi Keih Bahri	645.00	10235.00	-762.00	9180.00	262.00	25530.00	-1276.00	17083.00	4777.00	10311.80	7598.58
Delta	Gash Barka	3,526.00	67,811.00	-36,496.00	41,945.00	2,368.00	67,844.00	-1,225.20	90,880.00	21,006.00	53,468.80	31112.76
Delta	Maekel	476.00	173.00	106.00	654.00	320.00	197.00	85.40	487.00	348.00	108.20	295.46
Delta	Semenawi Keih Bahri	27356.00	991652.00	-471640.00	514045.00	16083.00	1006261.00	11133.60	1243787.00	255058.00	748808.60	434254.43

.

Sub-regional aggregation

Table S 11. Changes in sub-regional ES supply potential mean values in 2019 with respect to 2015, for maximum literature. Emphasized the subregions with the highest (blue) and lowest (red) overall mean values.

			Changes in subregional suppy potential mean values in 2019 - Maximum literature values										
			a	b	c	d	e	f	g	h	i	j	
Zoba	Subzoba	Area (ha)	Crop provisioning	Grazed biomass	Wood	Water supply	Global climate regulation	Soil & sediment retention	Pollination	Nursery population & habitat	Recreation-related	Spiritual, artistic & symbolic	Overall
Anseba	Adi Tekeliezan	37958.9	0.00	0.00	0.00	0.00	0.00	0.00	0.00	0.00	0.00	0.00	0.00
	Asmat	278819.0	0.01	0.27	-0.17	0.14	0.00	0.20	0.00	0.34	0.07	0.20	0.11
	Elabered	62770.7	0.00	0.00	0.00	0.00	0.00	0.00	0.00	0.00	0.00	0.00	0.00
	Geleb	43898.5	0.00	0.00	0.00	0.00	0.00	0.00	0.00	0.00	0.00	0.00	0.00
	Habero	150031.0	0.00	0.15	-0.10	0.08	0.00	0.10	0.00	0.19	0.04	0.11	0.06
	Hagaz	103691.2	0.00	0.00	0.00	0.00	0.00	0.00	0.00	0.00	0.00	0.00	0.00
	Halhal	79339.3	0.05	0.07	-0.04	0.05	0.02	0.03	0.01	0.08	0.01	0.06	0.03
	Hamelmalo	43090.6	0.00	0.00	0.00	0.00	0.00	0.00	0.00	0.00	0.00	0.00	0.00
	Keren	9196.6	0.00	-0.01	0.00	0.00	0.00	0.00	0.00	-0.01	0.00	-0.01	0.00
	Kerkebet	364314.2	0.01	0.15	-0.08	0.09	0.00	0.16	0.00	0.20	0.04	0.12	0.07
	Sel'a	927199.1	0.00	0.42	-0.21	0.21	0.00	0.42	0.00	0.52	0.10	0.31	0.18
Debub	Adi Keih	71668.9	0.00	0.00	0.00	0.00	0.00	0.01	0.00	0.01	0.00	0.00	0.00
	Adi Kuala	70543.3	0.00	0.00	0.00	0.00	0.00	0.00	0.00	0.00	0.00	0.00	0.00
	Areza	127933.9	0.00	0.00	0.00	0.00	0.00	0.00	0.00	0.00	0.00	0.00	0.00
	Dbarwa	95555.5	0.00	-0.01	0.00	0.01	0.00	0.00	-0.01	0.00	0.01	0.00	0.00
	Dekemhare	69368.0	0.00	0.00	0.00	0.00	0.00	0.00	0.00	0.00	0.00	0.00	0.00
	Emni Haili	41238.4	0.00	0.00	0.00	0.00	0.00	0.00	0.00	0.00	0.00	0.00	0.00
	Mai Aini	79117.6	0.00	0.00	0.00	0.00	0.00	0.00	0.00	0.00	0.00	0.00	0.00
	Mai Mne	100851.7	0.00	0.00	0.00	0.00	0.00	0.00	0.00	0.00	0.00	0.00	0.00
	Mendefera	62705.6	0.00	0.00	0.00	0.00	0.00	0.00	0.00	0.00	0.00	0.00	0.00
	Segeneiti	61440.5	0.00	0.00	0.00	0.00	0.00	0.00	0.00	0.00	0.00	0.00	0.00
	Senafe	108585.7	0.01	0.00	0.01	0.01	0.01	0.01	0.00	0.00	0.00	0.00	0.00
	Tsorona	77275.4	0.00	0.00	0.00	0.00	0.00	0.00	0.00	0.00	0.00	0.00	0.00
Debubawi Keih Bahri	Ara'eta	727434.2	0.00	0.02	0.00	0.01	0.00	0.04	0.00	0.02	0.01	0.01	0.01
	Assab	38290.2	0.01	0.01	0.00	0.01	0.00	0.01	0.00	0.01	0.00	0.01	0.01
	Central SRS	441673.4	0.00	0.00	0.00	0.00	0.00	0.00	0.00	0.00	0.00	0.00	0.00
	Southern SRS	1069818.5	0.00	0.00	0.00	0.00	0.00	0.00	0.00	0.00	0.00	0.00	0.00
Gash Barka	Akurdet	35307.3	0.00	0.04	-0.02	0.02	0.00	0.04	0.00	0.06	0.01	0.03	0.02
	Barentu	32911.5	0.00	0.00	0.00	0.00	0.00	0.00	0.00	0.00	0.00	0.00	0.00
	Dge	372884.4	0.01	0.02	-0.01	0.02	0.00	0.03	0.00	0.04	0.01	0.02	0.01
	Forto	454560.5	0.00	0.10	-0.05	0.05	0.00	0.10	0.00	0.13	0.03	0.08	0.04
	Gonei	258440.4	0.00	0.00	0.00	0.00	0.00	0.00	0.00	0.00	0.00	0.00	0.00
	Haikota	222346.1	0.00	0.08	-0.04	0.04	0.00	0.06	0.00	0.10	0.02	0.06	0.03
	La'elay Gash	437655.9	0.00	0.00	0.00	0.00	0.00	0.00	0.00	0.01	0.00	0.00	0.00
	Logo Anseba	62132.8	0.00	0.00	0.00	0.00	0.00	0.00	0.00	0.00	0.00	0.00	0.00
	Mensura	287897.4	0.00	0.00	0.00	0.00	0.00	0.00	0.00	0.00	0.00	0.00	0.00
	Mogolo	182777.3	0.00	0.00	0.00	0.00	0.00	0.00	0.00	0.00	0.00	0.00	0.00
	Molqi	234140.1	0.00	0.00	0.00	0.00	0.00	0.00	0.00	0.00	0.00	0.00	0.00
	Omhajer	506671.4	0.00	0.00	0.00	0.00	0.00	0.00	0.00	0.01	0.00	0.00	0.00
	Shambqo	169369.1	0.00	0.00	0.00	0.00	0.00	0.00	0.00	0.00	0.00	0.00	0.00
	Tesseney	110466.2	-0.01	0.06	-0.03	0.03	0.00	0.05	0.00	0.07	0.02	0.04	0.02
Maekel	Berik	27041.6	0.00	0.00	0.00	0.00	0.00	0.00	0.00	0.00	0.00	0.00	0.00
	Gala Nefhi	38783.9	0.00	-0.01	0.00	0.00	0.00	0.00	0.00	0.00	0.00	0.00	0.00
	Northern Asmara	711.9	0.00	0.00	0.00	0.00	0.00	0.00	0.00	0.00	0.00	0.00	0.00
	Northern Merab	3960.1	0.00	0.00	-0.01	0.01	0.00	0.00	-0.01	0.01	0.01	0.00	0.00
	Serejeqa	28463.2	0.02	0.02	0.00	0.02	0.01	0.01	0.01	0.02	0.01	0.01	0.01
	Southern Asmara	2591.0	0.00	0.00	0.00	0.00	0.00	0.00	0.00	0.00	0.00	0.00	0.00
	Southern Merab	2502.9	0.00	-0.01	0.00	0.00	0.00	0.00	-0.01	0.00	0.00	-0.01	0.00
Semenawi Keih Bahri	Adobhe	293101.1	0.00	0.06	-0.03	0.03	0.00	0.07	0.00	0.08	0.02	0.05	0.03
	Afabet	718527.1	0.01	0.56	-0.27	0.28	0.00	0.57	0.00	0.70	0.14	0.42	0.24
	Foro	269813.5	0.00	0.07	-0.03	0.03	0.00	0.07	0.00	0.09	0.02	0.05	0.03
	Gel'alo	707747.2	0.00	0.01	0.00	0.01	0.00	0.01	0.00	0.01	0.00	0.01	0.00
	Gindae	200104.6	0.08	0.07	0.06	0.11	0.06	0.09	0.05	0.11	0.06	0.07	0.08
	Massawa	32635.8	-0.08	-0.02	0.00	-0.03	-0.03	0.03	-0.02	0.00	0.01	-0.02	-0.02
	Nakfa	309023.3	0.00	0.39	-0.22	0.19	0.00	0.34	0.00	0.48	0.10	0.29	0.16
	Quarura	669355.4	0.00	0.64	-0.31	0.32	0.00	0.67	0.00	0.81	0.16	0.48	0.28
	Sheb	193439.8	0.06	0.92	-0.44	0.48	0.02	0.92	0.01	1.14	0.22	0.70	0.40

Table S 12. Changes in sub-regional ES supply potential mean values in 2019 with respect to 2015, for average literature. Emphasized the subregions with the highest (blue) and lowest (red) overall mean values.

			Changes in subregional suppy potential mean values in 2019 - Average literature values										
			a	b	c	d	e	f	g	h	i	j	
Zoba	Subzoba	Area (ha)	Crop provisioning	Grazed biomass	Wood	Water supply	Global climate regulation	Soil & sediment retention	Pollination	Nursery population & habitat	Recreation-related	Spiritual, artistic & symbolic	Overall
Anseba	Adi Tekeliezan	37958.9	0.00	0.00	0.00	0.00	0.00	0.00	0.00	0.00	0.00	0.00	0.00
	Asmat	278819.0	0.01	0.26	-0.09	0.04	0.03	0.14	-0.03	0.24	0.04	0.09	0.07
	Elabered	62770.7	0.00	0.00	0.00	0.00	0.00	0.00	0.00	0.00	0.00	0.00	0.00
	Geleb	43898.5	0.00	0.00	0.00	0.00	0.00	0.00	0.00	0.00	0.00	0.00	0.00
	Habero	150031.0	0.00	0.14	-0.06	0.02	0.01	0.07	-0.03	0.13	0.02	0.05	0.03
	Hagaz	103691.2	0.00	0.00	0.00	0.00	0.00	0.00	0.00	0.00	0.00	0.00	0.00
	Halhal	79339.3	0.04	0.05	-0.04	0.02	0.00	0.01	-0.02	0.06	0.00	0.01	0.01
	Hamelmalo	43090.6	0.00	0.00	0.00	0.00	0.00	0.00	0.00	0.00	0.00	0.00	0.00
	Keren	9196.6	0.00	-0.01	0.00	0.00	0.00	-0.01	0.00	-0.01	0.00	0.00	0.00
	Kerkebet	364314.2	0.01	0.15	-0.03	0.03	0.04	0.11	0.00	0.14	0.04	0.06	0.05
	Sel'a	927199.1	0.00	0.44	-0.08	0.05	0.12	0.31	0.00	0.37	0.08	0.16	0.14
Debub	Adi Keih	71668.9	0.00	0.00	0.00	0.00	0.00	0.00	0.00	0.00	0.00	0.00	0.00
	Adi Kuala	70543.3	0.00	0.00	0.00	0.00	0.00	0.00	0.00	0.00	0.00	0.00	0.00
	Areza	127933.9	0.00	0.00	0.00	0.00	0.00	0.00	0.00	0.00	0.00	0.00	0.00
	Dbarwa	95555.5	0.00	-0.01	0.00	0.01	0.00	0.00	0.00	0.00	0.01	0.00	0.00
	Dekemhare	69368.0	0.00	0.00	0.00	0.00	0.00	0.00	0.00	0.00	0.00	0.00	0.00
	Emni Haili	41238.4	0.00	0.00	0.00	0.00	0.00	0.00	0.00	0.00	0.00	0.00	0.00
	Mai Aini	79117.6	0.00	0.00	0.00	0.00	0.00	0.00	0.00	0.00	0.00	0.00	0.00
	Mai Mne	100851.7	0.00	0.00	0.00	0.00	0.00	0.00	0.00	0.00	0.00	0.00	0.00
	Mendefera	62705.6	0.00	0.00	0.00	0.00	0.00	0.00	0.00	0.00	0.00	0.00	0.00
	Segeneiti	61440.5	0.00	0.00	0.00	0.00	0.00	0.00	0.00	0.00	0.00	0.00	0.00
	Senafe	108585.7	0.00	0.00	0.01	0.00	0.01	0.01	0.01	0.00	0.00	0.00	0.00
	Tsorona	77275.4	0.00	0.00	0.00	0.00	0.00	0.00	0.00	0.00	0.00	0.00	0.00
Debubawi Keih Bahri	Ara'eta	727434.2	0.00	0.02	0.01	0.00	0.02	0.03	0.01	0.02	0.01	0.01	0.01
	Assab	38290.2	0.01	0.01	0.00	0.00	0.01	0.01	0.00	0.00	0.00	0.00	0.00
	Central SRS	441673.4	0.00	0.00	0.00	0.00	0.00	0.00	0.00	0.00	0.00	0.00	0.00
	Southern SRS	1069818.5	0.00	0.00	0.00	0.00	0.00	0.00	0.00	0.00	0.00	0.00	0.00
Gash Barka	Akurdet	35307.3	0.00	0.05	-0.01	0.01	0.01	0.03	0.00	0.04	0.01	0.02	0.02
	Barentu	32911.5	0.00	0.00	0.00	0.00	0.00	0.00	0.00	0.00	0.00	0.00	0.00
	Dge	372884.4	0.01	0.02	-0.01	0.02	0.01	0.02	0.00	0.03	0.01	0.01	0.01
	Forto	454560.5	0.00	0.10	-0.02	0.02	0.03	0.07	0.00	0.09	0.02	0.04	0.03
	Gonei	258440.4	0.00	0.00	0.00	0.00	0.00	0.00	0.00	0.00	0.00	0.00	0.00
	Haikota	222346.1	0.00	0.07	-0.02	0.01	0.01	0.05	0.00	0.07	0.01	0.03	0.02
	La'elay Gash	437655.9	0.00	0.00	0.00	0.00	0.00	0.00	0.00	0.00	0.00	0.00	0.00
	Logo Anseba	62132.8	0.00	0.00	0.00	0.00	0.00	0.00	0.00	0.00	0.00	0.00	0.00
	Mensura	287897.4	0.00	0.00	0.00	0.00	0.00	0.00	0.00	0.00	0.00	0.00	0.00
	Mogolo	182777.3	0.00	0.00	0.00	0.00	0.00	0.00	0.00	0.00	0.00	0.00	0.00
	Molqi	234140.1	0.00	0.00	0.00	0.00	0.00	0.00	0.00	0.00	0.00	0.00	0.00
	Omhajer	506671.4	0.00	0.00	0.00	0.00	0.00	0.00	0.00	0.00	0.00	0.00	0.00
	Shambqo	169369.1	0.00	0.00	0.00	0.00	0.00	0.00	0.00	0.00	0.00	0.00	0.00
	Tesseney	110466.2	-0.01	0.06	-0.01	0.01	0.01	0.04	0.00	0.05	0.01	0.02	0.02
Maekel	Berik	27041.6	0.00	0.00	0.00	0.00	0.00	0.00	0.00	0.00	0.00	0.00	0.00
	Gala Nefhi	38783.9	0.00	-0.01	0.00	0.00	0.00	0.00	0.00	0.00	0.00	0.00	0.00
	Northern Asmara	711.9	0.00	0.00	0.00	0.00	0.00	0.00	0.00	0.00	0.00	0.00	0.00
	Northern Merab	3960.1	0.00	0.00	0.00	0.01	0.00	0.00	0.00	0.01	0.00	0.00	0.00
	Serejeqa	28463.2	0.01	0.01	0.00	0.01	0.01	0.01	0.01	0.02	0.01	0.00	0.01
	Southern Asmara	2591.0	0.00	0.00	0.00	0.00	0.00	0.00	0.00	0.00	0.00	0.00	0.00
	Southern Merab	2502.9	0.00	-0.01	0.00	0.00	0.00	-0.01	0.00	0.00	0.00	0.00	0.00
Semenawi Keih Bahri	Adobhe	293101.1	0.00	0.07	-0.01	0.01	0.02	0.05	0.00	0.05	0.01	0.02	0.02
	Afabet	718527.1	0.01	0.59	-0.11	0.07	0.16	0.43	0.00	0.49	0.11	0.21	0.20
	Foro	269813.5	0.00	0.07	-0.01	0.01	0.02	0.06	0.00	0.06	0.02	0.03	0.03
	Gel'alo	707747.2	0.00	0.01	0.00	0.00	0.00	0.01	0.00	0.01	0.00	0.00	0.00
	Gindae	200104.6	0.02	0.04	0.08	0.04	0.07	0.08	0.07	0.07	0.05	0.03	0.05
	Massawa	32635.8	-0.07	0.01	0.02	-0.01	0.02	0.04	0.01	0.00	0.02	0.01	0.00
	Nakfa	309023.3	0.00	0.39	-0.11	0.05	0.07	0.25	-0.03	0.34	0.06	0.13	0.12
	Quarura	669355.4	0.00	0.68	-0.11	0.08	0.19	0.50	0.01	0.56	0.13	0.25	0.23
	Sheb	193439.8	0.05	0.96	-0.18	0.13	0.26	0.68	0.01	0.80	0.18	0.34	0.32

Section 4 - Links with ongoing national initiatives

In the following, three MAES-relevant ongoing initiative in Eritrea. The selected of examples refer to both the national and regional level; we thus want to highlight some of their impacts as well as possible links with a MAES application such as the one proposed in this study. The examples represent a good starting point for exploring potential causal links between identified ecosystem changes and current policies, and projects implemented on the ground, possibly involving key actors dealing with biodiversity and ES issues in the country. By way of example, Box 1 offers some insights considering some large dam project as well as protected areas.

Example 1

Greening Campaigns to restore Ecosystems in Eritrea

Since 1991, the Government of Eritrea (GoE) has taken initiatives to enhance the country's biodiversity by adopting mechanisms and mobilizing communities and resources. These activities are now undertaken by the Forest and Wildlife Authority (FWA). Overall, the impact of all the ecosystem restoration initiatives is significant. Among other things, 132 million trees have been planted in the last 29 years, or an average of 4.6 million trees per year. In addition, the FWA has established a total of 396,930 hectares of protected area, out of about **two million hectares** planned to be enclosed in the future, to promote natural regeneration of trees and grasses. The enclosures have flourished in biodiversity and many endangered wildlife are returning to the habitat while new ones are emerging. In all this, the participation of people from all walks of life, especially members of the defense forces, students and local communities, in afforestation and reforestation programs has been crucial. Notably, more than 500 Green Clubs have been established across; yet, only 50% of the planned number of clubs have been created. This is also indicative of the arduous challenges ahead.

(**Source**: Habtom Tesfamichael, Ministry of Information, 30/03/2022; Link, last accessed 01/05/2022).

Example 2

Maekel Region: Success in Reforestation

According to Wildlife and Forestry Authority in the Maekel region, reforestation activities have been taken place for over 28 year, with the involvement of all sectors of society, including students participating in Summer Work Programs. Of note, through it four major, and five smaller nurseries, the region has been provided 1 million seedlings every year. These include eucalyptus and Indigenous tree species, the former being the most widely preferred tree in the highlands (by the inhabitants) because it grows fast and has big demand in the construction sector. The region can serve as a living lab to advance MAES application in the country and thus contribute to the ambitious greening campaign in the country.

(**Source**: Kesete Ghebrehiwet, Ministry of Information, 07/04/2022; Link, last accessed 01/05/2022).

Example 3

The construction of small and large dams in Eritrea
Since independence in 1991, some 785 large and small dams have been built with local resources amounting to several millions of US dollars, that have contributed significantly to ensuring water and food security in the country. These include 557 small dams (i.e., 10K to 100K m^3), 198 medium dams (i.e., 100K to 1M m^3), and large (i.e., 1M to 330M m^3) dams distributed in the six regions. Specifically, there are some 164 small and micro dams in the Zoba Anseba, 338 in the Zoba Debub, 145 in Zoba Gash Barka, 115 in the Zoba Maekel, and 23 in the Semenawi Keih Barhi region. Table S 13 presents and overview of the major dams built since 2003.

Table S 13. Major dams built since 2003

Dam name	Region	Capacity (m^3)	Construction	Cost (NKF)	Company
Kerkebet	Gash-Barka	330 million	2009-2013	1 Billion	Red Sea & Bidho
Gahtelai	Semenawi Keih Bahri	50 million	2016	-	Gedem
Misilam (Gherghera)	Debub	36 million	2010-2014	-	
Logo Dam	Maekel - Debub	31 million	2013	-	
Bademit Dam	Gash-Barka	32 million	2011-2012	-	Bidho
Gerset Dam	Gash-Barka	20 million	2005 - 2009	590 Million	Segen
Fanco-Rawi Dam	Gash-Barka	20 million	2008 - 2011	255 Million	Segen
Fanco-Tsimu	Gash-Barka	14 million	2003-2006	334 Million	Segen

*Box S 1. Three examples of dam projects as entry point to explore the application of MAES results. Satellite images representing the condition before and after construction of major dams: A) **Kerkebet Dam**; B) **Gerset and Fanko Dam**; and C) **Logo Dam** located in Gala Nefhi sub-region in Zoba Maekel.*

Ecosystem changes due to dam construction and SLM interventions	Insight for links to our results

A

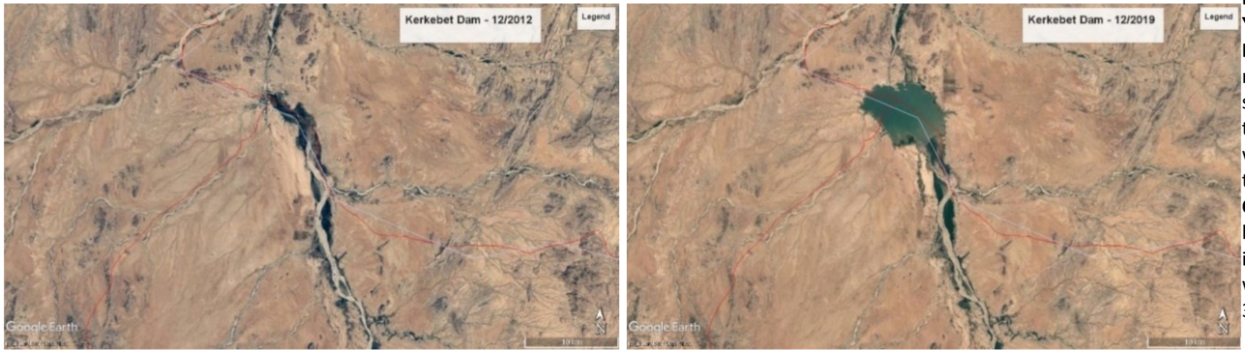

Kerkebet Dam
Years: 2012-2019
Location: Kerkebet subregion in Zoba Anseba neighboring Dge (to southwest) and Forto (northwest) sub-regions in Zoba Gash-Barka. Completed in 2013, it is the largest in the country built along the Barka River with the aim of increasing water resources and changing the surrounding ecosystems.
Our results: permanent water bodies increased by 0.358 km² (+5.9%) in Kerkebet subregion, by 0.131 km² (+2.3% in Dge, and 0.036 km2 (+1.3%) in Forto; herbaceous wetland increased by 1.338 km2 (+187%) in Kerkebet, by 3.175 km2 (+321%) in Dge, and 0.036 km² in Forto

B

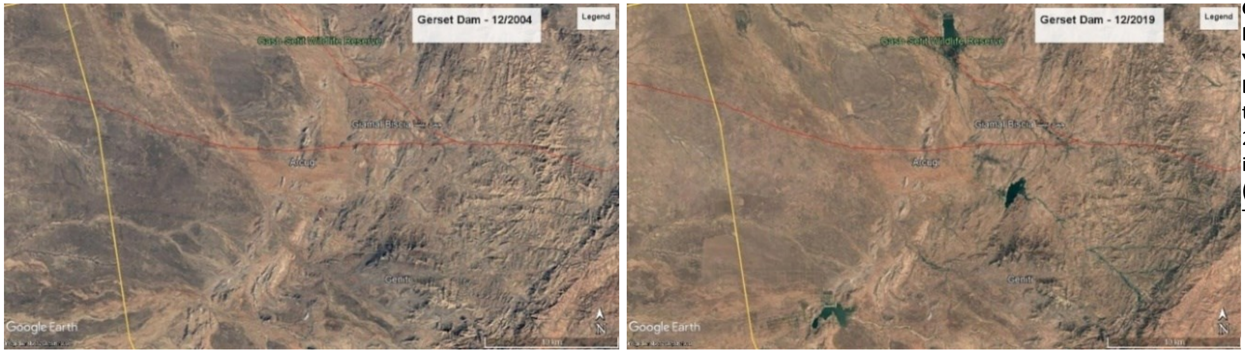

Gerset Dam and Fanko Dam and the Gash Setit Wildlife Reserve.
Years: 2004-2019
Location: Omhajer, Tesseney, and Haikota subregions of the Zoba Gash Barka, **Our results:** over the period 2015-2019, Herbaceous wetland in Gash-Bark subregions have increased by 0.036 km2 (60%) in Hikota, 0,358 Km2 (+150%) in Omhajer, and by 0,119 km2 (+333%) in Tesseney.

C

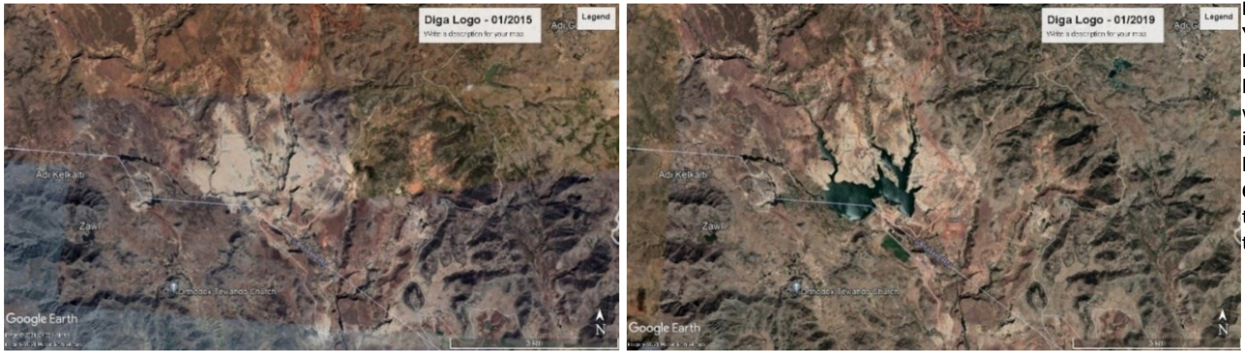

Logo Dam
Years: 2015-2019
Location: Gala Nefhi sub-region in Zoba Maekel, bordering Zoba Debub, is a flagship project related to water and food security, social services provision and industrialization, in the densely populated Eritrean highlights.
Our results: According to our findings, water bodies in the sub-region increased by 3,6 ha or by 13% from 2015 till 2019.
